# Microglia in diffuse midline glioma contribute to extracellular matrix remodelling and cancer cell invasion

**DOI:** 10.1038/s41419-026-08891-y

**Published:** 2026-05-30

**Authors:** Lily Keane, Martin Škandík, Mercedes Posada-Pérez, Raj Bose, John Desito, Esmee van der Linde, Pinelopi Engskog-Vlachos, Sandra Ceccatelli, Adam L. Green, Bertrand Joseph

**Affiliations:** 1 Institute of Environmental Medicine, Toxicology unit, Karolinska Institutet, Stockholm, Sweden; 2 Center for Neuromusculoskeletal Restorative Medicine, Hong Kong Science Park, Shatin, Hong Kong, China; 3 Department of Neuroscience, Karolinska Institutet, Stockholm, Sweden; 4 Morgan Adams Foundation Pediatric Brain Tumor Research Foundation, Department of Pediatrics, University of Colorado Anschutz Medical Campus Aurora, Aurora, CO USA; 5 Present Address: APC Microbiome Ireland, University College Cork, Cork, Ireland; 6 Present Address: Department of Anatomy & Neuroscience, University College Cork, Cork, Ireland; 7 Present Address: Ribocure Pharmaceuticals AB, Gothenburg, Sweden

**Keywords:** CNS cancer, Microglial cells

## Abstract

Diffuse midline glioma, H3K27-altered (DMG), is an aggressive and uniformly fatal paediatric brain tumour arising in midline structures and characterised by substantial microglial infiltration. We investigated whether microglia adopt a reactive state in response to DMG cells that functionally contributes to tumour progression. Transcriptomic profiling of microglia exposed to DMG, H3K27M cells, together with analysis of tumour associated myeloid cells isolated from DMG patient biopsies, revealed a pronounced upregulation of extracellular matrix (ECM) components, including fibronectin. Single cell transcriptomic analysis further identified microglia as the primary fibronectin expressing cell population within human DMG, H3K27M tumours. Functional invasion assays using a panel of patient-derived DMG, H3K27M cells, revealed that microglia-derived fibronectin significantly enhances tumour cell invasiveness, while its chemical inhibition with RGDS peptide or Avapritinib or its genetic silencing using small-interfering RNAs effectively suppresses invasion. Across independent patient cohorts (Kids First, PNOC, and CBTTC), and in archival tissues, DMG tumours were found to exhibit elevated expression of ECM components, and high fibronectin expression that correlated with poor prognosis. These findings suggest that microglia actively contribute to DMG invasiveness through ECM component production, identifying fibronectin as a potential therapeutic target in this lethal paediatric cancer.

## Introduction

Diffuse midline glioma, H3K27-altered (DMG), classified within the family of paediatric-type diffuse high-grade gliomas (pHGG), is an aggressive tumour that arise in midline structures of the brain, most commonly the brainstem, thalamus and spinal cord [1]. When located in the pons area of the brainstem these tumours are also known as diffuse intrinsic pontine glioma (DIPG). DMG is reported to account for up to 20% of all childhood brain tumours, with most cases occurring in children aged 5–9 years old [2]. These neoplasms are the leading cause of brain tumour-related death in children, with a median survival time from diagnosis being less than one year, and a 5-year overall survival around 2% [3, 4]. The remaining pHGG are diffuse hemispheric glioma H3G34-mutant, diffuse paediatric-type high-grade glioma H3-wildtype and IDH-wildtype, and infant-type hemispheric glioma, which primarily occur in the cortical hemispheres. They exhibit a slightly better prognosis as compared to DMG with a median overall survival ranging from 14 to 44 months depending on their molecular alterations [5].

The midline region of the brain is involved in many of the body’s most vital functions, and thereby the DMG tumour location implies limitations for interventions. Currently, palliative radiation remains the standard therapy for DMG to halt progression and reduce tumour bulk to minimize symptoms. However, radio-resistance ultimately arises in all DMG, resulting in tumour recurrence and a fatal outcome for the patients [6]. Hence, there is an urgent need to develop new therapeutic strategies. In fact, the biology of DMG cancer cells is becoming better characterized, and a few products harnessing these discoveries are under development. The most promising therapies include dordaviprone (ONC201, dopamine receptor D2 antagonist and an allosteric activator of the mitochondrial caseinolytic protease P), as well as immunotherapies such as CAR-T cells targeting the disialoganglioside GD2 expressed at high levels by DMG; which have both entered clinical trials [7–9]. However, to date they only extend lifespan by months and therefore new treatment options are needed.

DMG, H3K27-altered are defined by a loss of histone H3p.K28me3 (K27me3, lysine-27 trimethylation) expression. Trimethylation of lysine 27 on H3 histones is mediated by the methyltransferase catalytic unit, enhancer of zeste homolog 2 (EZH2), of the Polycomb Repressive Complex 2 (PRC2). DMG are divided into subtypes based on the genetic alteration that contribute to the widespread loss of K27me3: H3 c.83A>T p.K28M (lysine-27 to methionine, K27M) substitution in one of the histone H3 isoforms encoding genes (mostly, *H3F3A* (for H3.3), and *HIST1H3B* (for H3.1)) for H3K27-mutant subtypes, aberrant overexpression of EZH inhibitory protein (EZHIP) for the H3-wildtype with EZHIP overexpression subtype or mutation or amplification of epidermal growth factor receptor (*EGFR*) for the *EGFR*-mutant subtype [10].

Reported independently by several research teams, H3.1K27M and H3.3K27M mutations were found to be molecular characteristics of DMG that hold robust prognostic value [11–13]. Thereafter, these somatic mutations were found to be associated to epigenetic-mediated chromatin remodelling and unique gene expression profiles [10, 14]. Preclinical murine models of the disease, encompassing patient-derived orthotopic xenograft in immunodeficient mice, genetically engineered mouse models based on the use of RACS/tv-a, SB/PiggyBac transposon with lentivirus, and Nestin-Cre/LoxP recombination systems, as well as immunocompetent syngeneic allograft mouse models, have all shown that H3K27-alterations including H3K27M mutations, potentiate DMG tumour development in mice [15–18]. H3K27-alterations are established as drivers of DMG tumorigenesis as well as their resistance to treatments, but additional drivers should contribute to the invasive growth of DMG tumours.

Beyond H3K27-alterations, DMG tumours are also characterised by a unique immunologically cold tumour microenvironment which contributes to disease progression [19–21]. The DMG tumour microenvironment is fundamentally different than adult-type diffuse gliomas such as glioblastoma. The comparative analysis of immunocompetent de novo mouse models of pHGG, inducing H3.3WT and H3.3G34R pHGG in the cortex, as well as H3.3WT, H3.1K27M, and H3.3K27M DMG in the midline revealed that the driver mutations and anatomical locations together uniquely shape the immune infiltrate. H3.3K27M DMG, demonstrated the highest enrichment of myeloid cells [22]. Accumulating evidence indicates that human DMG tumours, similar to murine DMG tumours, present a substantial infiltration of tissue-resident microglia as well as peripherally recruited bone marrow-derived macrophages but limited T lymphocyte or NK cells infiltration [17, 23, 24]. Single cell RNA-sequencing (RNA-seq) analyses performed on fresh DMG biopsies indicate that myeloid cells in the tumour mass are negative for H3K27M [25]. We previously confirmed that in the context of DMG tumours carrying a H3K27M mutation, microglia within the tumour microenvironment are wildtype for the histone H3 [26]. We also reported that by inducing a loss of H3K27me3 in microglia, DMG cancer cells push microglia toward a tumour-supporting reactive state that and in turn promote DMG cancer cells migration and invasion capabilities [26]. Here we further investigate the impact DMG cancer cells have on the transcriptomic response of microglia and intended to uncover microglial factors that could mediate their tumour trophic effects.

## Results

### Increased presence of reactive microglia in human H3K27M DMG

**Fig. 1:**
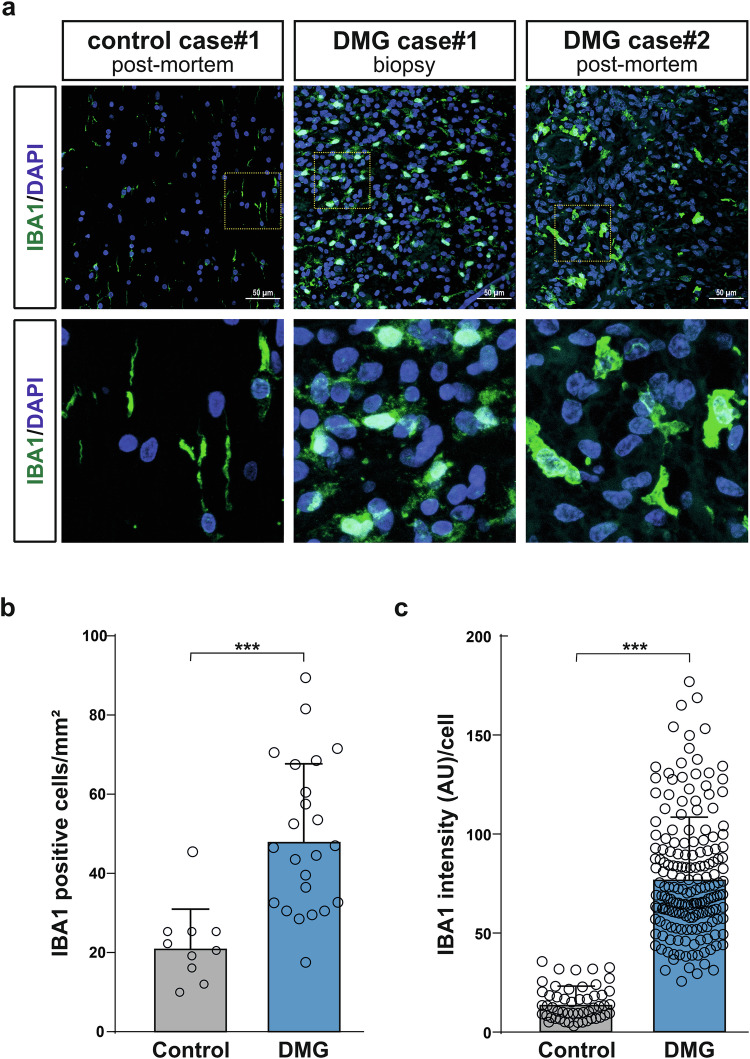
Increased presence of reactive microglia in human H3K27M DMG tumours. **a** Confocal microscopy imaging of two human DMG tumours (one biopsy and one post-mortem samples), and one age-matched pons control case (post-mortem sample), with immunofluorescence staining for IBA1. DAPI used as nuclear counterstain, scale bars (50 µm). **b** IMARIS quantification of the numbers of IBA1-positive cells/mm^2^, and (**c**), IMARIS quantification of IBA1 fluorescence intensity/cell from the archival tissues described in panel a. Data are mean ± SEM from independently analyzed microglia from 2 DMG (1 biopsy, 1 post-mortem) and 1 age-matched control human tissues. Statistical annotation ***p < 0.001; for indicated comparisons. See Suppl. Fig. 1, for additional two human DMG tumours, and one age-matched pons control case.

We previously reported that microglia, wildtype for histone H3, are present in the tumour mass from DMG H3K27M subjects and that in vitro microglia exposed to patient-derived DMG cancer cells acquired a reactive state holding tumour trophic functions [26]. The presence of microglia was further analysed in archival human DMG tissue samples obtained from the BRAIN UK biobank. A total of four DMG cases, with reported H3K27M mutation, were examined, including three biopsies taken at the time of diagnosis and one post-mortem sample. These DMG cases were compared to two age-matched brainstem control cases, in which the individuals had died from non-neurological causes (Suppl. Table 1). Microglial numbers and activation states were assessed using IBA1 (ionized calcium binding adaptor molecule 1, also known as allograft inflammatory factor 1, AIF1) immunofluorescence staining [27, 28]. The paediatric brain DMG tissue samples exhibited an increased presence of microglia as compared to the control pons tissue samples (Fig. 1a, b; Suppl. Fig. 1). Additionally, an increased fluorescence intensity of IBA1 per cell was observed in the DMG cases (Fig. 1a, c; Suppl. Fig. 1). Collectively, our previous findings and these observations support the notion that microglia play an active role in DMG tumour progression rather than being passive bystanders.

### Microglia exposed to DMG H3K27M cells exhibit a transcriptomic response associated with extracellular matrix remodelling

**Fig. 2:**
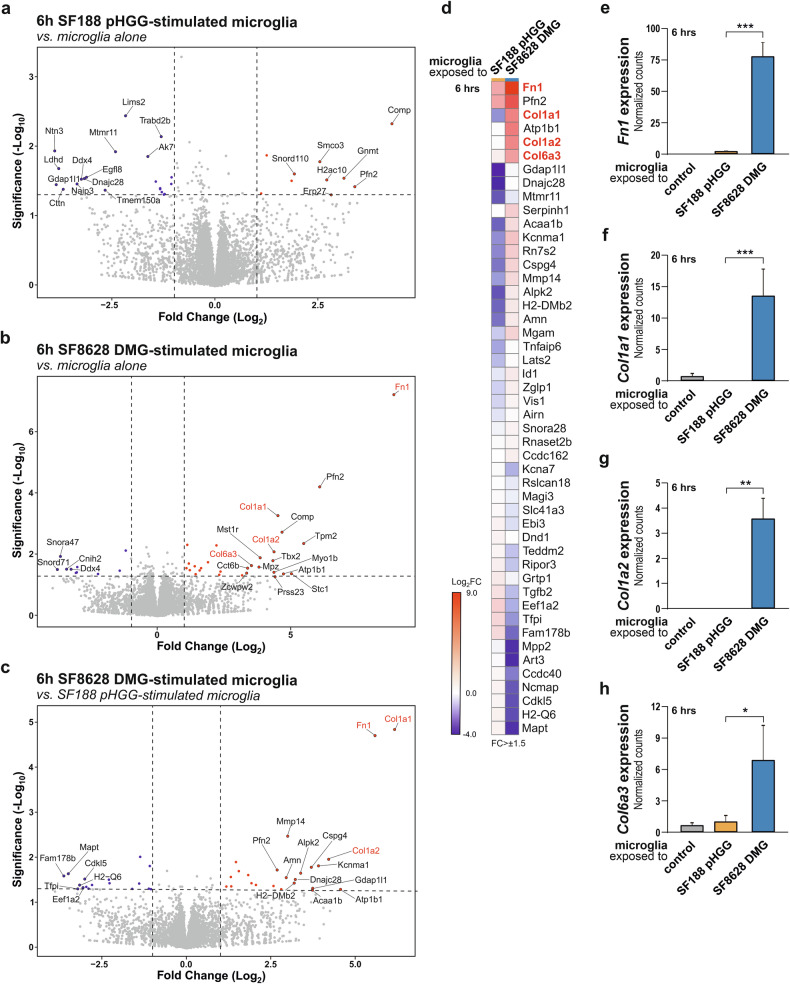
Microglia exposed to DMG H3K27M cells and pHGG cells exhibit different transcriptomic responses. **a**, **b** Volcano plots illustrating differentially expressed genes (DEGs) based on the log_2_(fold change) related to negative log_10_ (*P*-value) between microglia exposed to SF188 pHGG cells for 6 h and microglia alone (**a**) or between microglia exposed to SF8628 DMG cells for 6 h and microglia alone (**b**). **c** Volcano plots illustrating DEGs between 6-hour SF8628 DMG cells-stimulated microglia compared to microglia alone and 6-hour SF188 pHGG cells-stimulated microglia compared to microglia alone. Blue dots represent significantly downregulated genes with log_2_(FC) of maximally -1, and red dots significantly upregulated genes with log_2_(FC) at least 1. Names of the top 20 genes for up- or downregulated genes are depicted. **d** Heatmap representation of genes found to be statistically differentially expressed (FC > 1.5) between conditions described in (**c**). Expression data for these genes were extracted from the comparisons of microglia exposed for 6 hours to SF188 pHGG cells compared to microglia alone or SF8628 DMG cells compared to microglia alone. **e**–**h** *Fn1*, *Col1a1*, *Col1a2* and *Col6a3* gene expressions as normalized counts, presented as mean ± SEM, in microglia exposed to SF188 pHGG cells for 6 h, microglia exposed to SF8628 DMG cells for 6 h and microglia alone. Data depicted in this figure originate from RNA-seq analysis of 3 independent biological replicates for each group. Statistical annotations *p < 0.05; **p < 0.01; and ***p < 0.001; for indicated comparisons. See Suppl. Fig. 2, for similar analysis on a 3-hour time point.

To gain further insights into the reactive state acquired by microglia when exposed to DMG cancer cells and how this pro-tumoral microglial phenotype could contribute to DMG progression, their transcriptomic response was investigated. For these experiments, a segregated co-culture system based on soluble factor exchange was used [26, 29, 30]. BV-2 microglia [31] were exposed to primary human cancer cells originating from a 3-year old female diagnosed with DIPG, SF8628, carrying the H3K27M mutation (thereafter referred as SF8628 DMG cells) [32, 33] or to SF188 cells representing pHGG originating from a 8-year old male right frontal lobe glioblastoma which harbour a H3-wildtype (H3-wt) status (referred as SF188 pHGG cells [34, 35]. Microglia exposed to SF8628 DMG cells or SF188 pHGG cells, for 3 and 6 h, were collected, and bulk RNA-seq was performed on three independent biological replicates (Fig. 2; Suppl. Fig. 2). Volcano plots illustrate the most significantly up- and down-regulated differentially expressed genes (DEGs) in microglia exposed to SF188 pHGG cells or SF8628 DMG cells for 3 h (Suppl. Fig. 2a, b) and 6 hours (Fig. 2a, b), as compared to microglia alone (Fold Change > 1.5 or < −1.5; *P*-value < 0.05). A direct comparison of the transcriptome from microglia exposed to SF8628 DMG cells compared to microglia alone, versus microglia exposed to SF188 pHGG cells compared to microglia alone revealed that microglia exposed to SF8628 DMG cells exhibited a significantly higher expression of genes related to the extracellular matrix (ECM) (Fig. 2c, d; Suppl. Fig. 2c, d). These ECM-related genes included *Fn1* (encoding for fibronectin), *Col1a1*, and *Col6a3* (collagens type I alpha 1 chain and type VI alpha 3 chain, respectively) at the 3-hour time point, as well as *Col1a2* (collagen type I alpha 2 chain) at the 6-hour time point. Normalized counts for the microglial expression of those ECM-related genes after 3- and 6-hour exposure to SF188 pHGG cells or SF8628 DMG cells and microglia alone (used as control) are presented in Fig. 2e–h; Suppl. Fig. 2e–h. Collectively, these transcriptomic data indicate that the response of microglia, resident immune cells of the brain, to DMG H3K27M cells is distinct from their response to pHGG H3-wt cells.

**Fig. 3:**
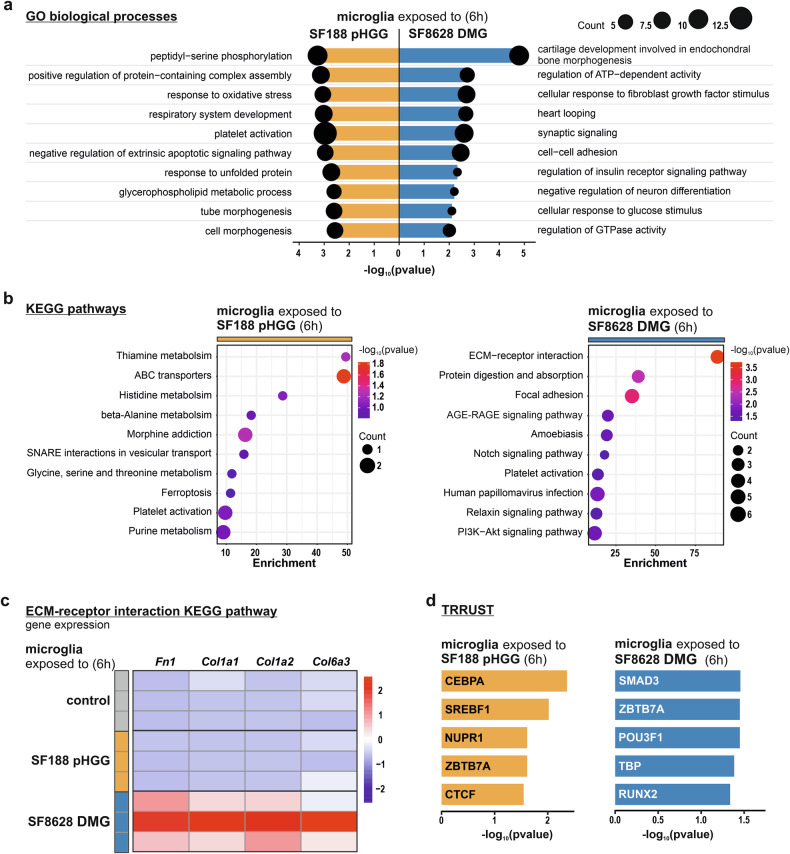
Microglia exposed to DMG H3K27M cells exhibit a transcriptomic response associated to extracellular matrix remodelling. **a** Analysis of enriched Gene Ontology (GO) terms for biological processes showing the top 10 significant terms sorted by -log_10_(p-value) for both, microglia exposed to SF188 pHGG cells and SF8628 DMG cells at 6-hour time point. Size of circle represent number of genes included in each GO BP term. **b** KEGG pathways enrichment analysis that allows identification of significantly affected pathways related to upregulated genes in SF188 pHGG cells-stimulated microglia and SF8628 DMG cells-stimulated microglia. The top 10 significant terms sorted by -log_10_(p-value) are displayed. Size of circles show number of genes included in each term and X-axis represent calculated enrichment. **c** Heatmap represent genes included in ECM-receptor interaction KEGG pathway displaying expression of genes of interest between experimental conditions. **d** TRRUST analysis, which allows the identification of potentially involved transcription factors based on the expression of their target genes in SF188 pHGG cells-stimulated microglia and SF8628 DMG cells-stimulated microglia. The top 5 genes sorted by -log_10_(*p*-value) are shown. Data depicted in this figure originate from RNA-seq analysis of 3 independent biological replicates for each group. See Suppl. Fig. 3, for similar analysis on a 3-hour time point.

Bioinformatic analysis revealed distinct Gene Ontology (GO) enrichment patterns for biological processes between microglia exposed to different tumour genetic backgrounds. GO biological process analysis showed that microglia exposed to pHGG cells express genes involved in immune activation and DNA damage repair at the 3-hour time point (Suppl. Fig. 3a). After 6-hour stimulation, the microglial response to pHGG cells shifted toward regulating protein function and assembly, with an increased expression of genes associated with stress responses (Fig. 3a). In contrast, microglia exposed to DMG cells exhibit an enrichment in biological processes related to cellular adhesion, morphogenesis, and regulation of cell movement, reflecting early tissue remodelling at the 3-hour time point (Suppl. Fig. 3a). After 6-hour stimulation, the microglial response to DMG cells shifted toward roles in developmental processes, synaptic signalling, together with engagement in metabolic and signalling transduction pathways (Fig. 3a). Similar results were obtained from Kyoto Encyclopedia of Genes and Genomes (KEGG) pathway analysis, revealing pathways linked to immune activation in microglia exposed to pHGG cells and extracellular matrix remodelling in microglia exposed to DMG cells at the 3-hour time point (Suppl. Fig. 3b). At the 6-hour time point, microglia exposed to pHGG cells displayed pathways associated with metabolic reprogramming, suggesting a shift toward supportive functions. Meanwhile, microglia exposed to DMG cells continued to exhibit pathways related to tissue remodelling (Fig. 3b). To further explore these findings, we investigated the expression patterns of genes involved in the “ECM receptor interaction” KEGG pathway, which was identified as a prominent hit in the DMG context at both time points. At 3 hours, the genes *Fn1, Col1a1, Col1a2, Col4a2, Col6a3*, and *Itga3* were expressed (Suppl. Fig. 3c), while at 6 h, *Fn1, Col1a1, Col1a2*, and *Col6a3* remained highly expressed (Fig. 3c). Heatmap representation for this KEGG pathway shows a significantly higher expression of these genes in microglia exposed to DMG cells as compared to microglia exposed to pHGG cells or microglia alone. Transcription factors and their selective target genes are central players in transcriptional regulation and in the acquisition of transcriptional programs controlling cell phenotypes. The transcription factor-target genes interaction database TRRUST (Transcriptional Regulatory Relationships Unravelled by Sentence-based Text mining), which allows for the identification of transcription factors potentially involved in cellular responses based on the expression of their target genes, further revealed that different sets of transcription factors are involved in the acquisition of the microglial reactive states observed upon their stimulation by DMG cells versus pHGG cells (Fig. 3d; Suppl. Fig. 3d). Hence, these transcriptomic data revealed that the responses of microglia to an exposure to pHGG cells and DMG cells are distinctive in term of their gene expression profile and that the microglial reactive states acquired upon stimulation by DMG cells is characterized by a further ECM-related transcripts enrichment.

### Inhibition of fibronectin derived from microglia reduces DMG H3K27M cell invasiveness

**Fig. 4:**
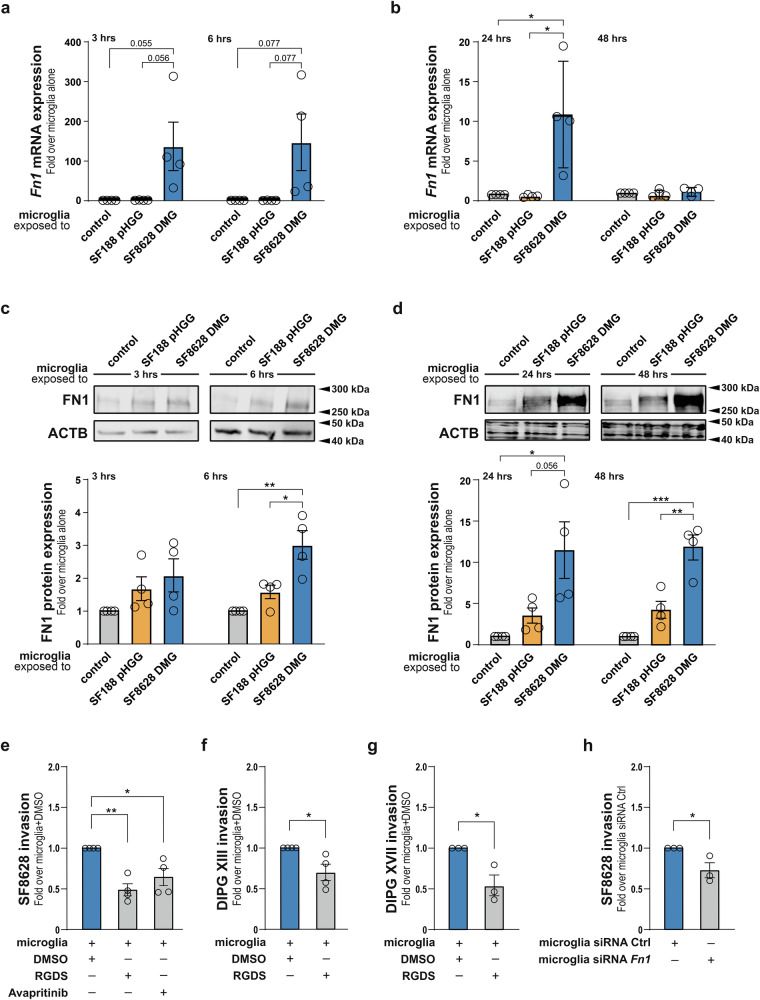
Inhibition of fibronectin derived from microglia reduces DMG H3K27M cell invasiveness. **a**, **b** RT-qPCR analysis confirm upregulation of *Fn1* mRNA expression in microglia exposed to SF8628 DMG cells, as compared to microglia exposed to SF188 pHGG cells or microglia alone (**a**) at 3- and 6-hour time point, (**b**) at 24- and 48-hour time point on. **c**, **d** Immunoblot analysis and quantification reveal upregulation of FN1 protein expression in SF8628 DMG cells-stimulated microglia, as compared to SF188 pHGG cells-stimulated microglia or microglia alone (**c**) at 3- and 6-hour time point, (**d**) at 24- and 48-hour time point. **e**–**g** Quantification of the DMG cells invasion capacity (**e**) SF8628 in presence of microglia and FN1 inhibitors RGDS and Avapritinib, (**f**) DIPG-XIII and (**g**) DIPG-XVII in presence of microglia and FN1 inhibitor RGDS. **h** Quantification of the effect of *Fn1* siRNA in microglia on SF8628 invasion capacity. Data are mean ± SEM from 4 (**a**–**f**) and 3 (**g**, **h**) independent biological replicates. Statistical annotations *p < 0.05; **p < 0.01; and ***p < 0.001; or exact p-value for indicated comparisons. See Suppl. Fig. 4, for *Col1*a1 mRNA and ICC protein expression validation and control experiments for the invasion assay.

The induction of *Fn1* and *Col1a1* gene expression in microglia exposed to SF8628 DMG cells, relative to both microglia exposed to SF188 pHGG cells and microglia alone, was confirmed by RT-qPCR analysis (Fig. 4a, b; Suppl. Fig. 4a), validating the upregulation of ECM-related factors identified from bulk RNA-seq (Fig. 2 and Suppl. Fig. 2). Consistent with these transcriptional changes, immunoblot analysis demonstrated a marked increase in FN1 protein levels in microglia exposed to SF8628 DMG cells for 6, 24 and 48 h, as compared those stimulated by SF188 pHGG or microglia alone (Fig. 4c, d). The COL1A1 protein levels showed only modest trend toward upregulation at the 3-hour time point, as assessed by immunoblot (Suppl. Fig. 4b). Therefore, we decided to validate COL1A1 protein expression by immunocytochemistry in microglia itself, exposed to SF188 pHGG cells or SF8628 DMG cells. Analysis of COL1A1 fluorescence intensity in microglia related to their size shows the same trends to mild upregulation at 3 h time point as immunoblot approach (Suppl. Fig. 4c, d).

Given that significant induction of FN1 expression was established as a feature of the response of microglia stimulated by DMG cancer cells, not observed in microglia stimulated by pHGG cancer cells, we decided to explore whether inhibiting this ECM component would alter tumour cell invasiveness.

The RGDS (Arg-Gly-Asp-Ser) tetrapeptide is a well-established antagonist of FN1 [36, 37]. This peptide blocks the integrin-binding domain, preventing tumour cell attachment to FN1. Although originally described as a PDGFRA inhibitor, Avapritinib was recently identified in a large-scale drug repurposing screen of 2471 FDA-approved compounds as a potent inhibitor of FN1 [38]. Avapritinib forms a more stable complex with FN1 than any other screened drug and induces substantial alterations in the secondary structure of the extra domain A (EDA) region of FN1, interfering with its function. Of importance for the present study, in vivo validation for the potential benefit of using Avapritinib in the context of DMG tumours was recently established. Indeed, Avapritinib treatment has been shown to significantly reduce DMG tumour growth in animal models of the disease, and a subset of paediatric and young adult *PDGFRA*-altered HGG patients showed clinical response to Avapritinib [39]. A key point for our investigation is that microglia do not express PDGFRA (based on the Human Protein Atlas, and observed absence of *PDFFRA* expression in microglia in the human DMG single-cell RNA-seq dataset described below), inferring that observed effects of Avapritinib on microglia cannot be attributed to PDGFRA inhibition but instead are most likely mediated via FN1 inhibition.

A mixture of SF8628 DMG cells and microglia treated with or without the RGDS peptide or Avapritinib embedded in Matrigel were placed on the upper compartment of a transwell migration assay, and the invasion capabilities of the DMG cells assayed. A significant reduction in tumour cell invasion in the presence of any of the FN1 inhibitors, as compared to controls, was observed for SF8628 DMG cells (Fig. 4e). RGDS and Avapritinib treatments were also shown to reduce the invasion capability of the microglia (in the presence of SF8628 DMG cells) but not of the SF8628 DMG cells alone (Suppl. Fig. 4e, h). The effect of RGDS peptide-treated microglia was also assessed on two additional H3K27M carrying DMG primary cell lines, i.e., DIPG-XIII and DIPG-XVII [40]. providing similar results with the exception that RGDS treatment on its own altered the invasion ability of DIPG XVII cells (Fig. 4f, g; Suppl. Fig. 4f, g).To directly assess whether microglial FN1 contributes to both microglial invasion and microglia-mediated promotion of tumour invasion, we silenced *Fn1* expression in microglia using a pool of small interfering RNAs (siRNAs). Efficient knockdown of *Fn1* significantly reduced the invasive capacity of microglia themselves and markedly diminished their ability to enhance SF8628 DMG cell invasion (Fig. 4h; Suppl. Fig. 4j).

Collectively, these results suggest that microglia-derived ECM components, particularly FN1, shape the tumour microenvironment and promote the invasive behaviour of DMG cells.

### Human DMG H3K27M tumours exhibit an increased expression of tumour-associated myeloid cell-derived ECM components

To gain pathophysiological understandings into the potential role for the uncovered microglial ECM-related factors in the DMG H3K27M tumour microenvironment, a meta-analysis was performed comparing the transcriptomic data generated from BV-2 microglia exposed to SF8628 DMG cells at 3- and 6-hour time points with an independent dataset generated from CD45^+^CD11b^+^ expressing cells isolated from human DMG tissue samples at the time of early post-mortem autopsy (Suppl. Fig. 5a). CD45^+^CD11b^+^ expression in normal paediatric tissue corresponds to the resident microglial population [24]. However, in the context of a brain neoplasm, CD45^+^CD11b^+^ expression does not allow the differentiation between resident microglia and peripherally recruited bone-marrow-derived macrophages, and therefore the analysed cell population should be considered as tumour-associated myeloid cells (TAMs, i.e., microglia and macrophages). This human dataset comprises six cases of DMG and three paediatric control samples derived from normal cortical tissue [24]. The comparative analysis of 3,825 DEGs (defined by a fold change > 2 or < −2, irrespective of statistical significance) from TAMs isolated from human DMG biopsies (as compared to control cases) and 1234 DEGs from microglia exposed to SF8628 DMG cells (as compared to microglia alone) at the 3 h time point, or 1254 DEGs at the 6-hour time point, revealed an overlap for 95 DEGs and 88 DEGs when the 3 h and 6 h time point were used, respectively (Suppl. Table 2).

**Fig. 5:**
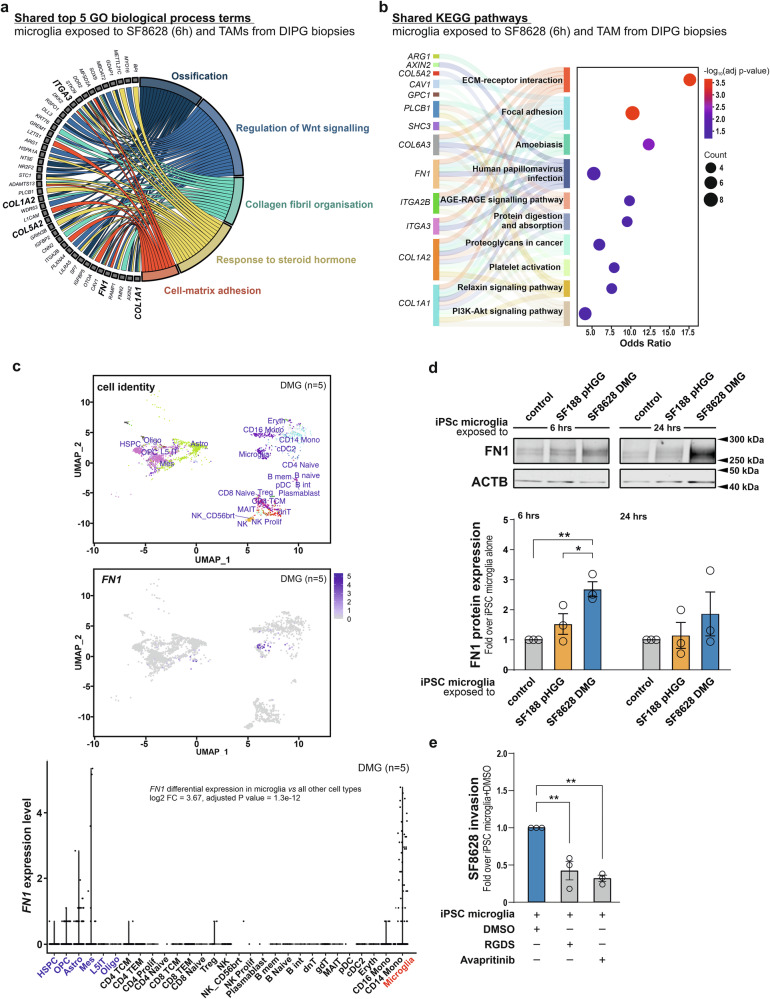
Tumour-associated myeloid cells exhibit expression of ECM-related genes in human DMG H3K27M tumours. **a** Chord diagram representing the top 5 shared GO biological process terms between in vitro assayed SF8628 DMG cells-stimulated microglia (6-hour time point) and TAMs from paediatric DMG with highlighted genes involved in extracellular matrix remodulation. **b** Sankey diagram combined with dot plot representing top 10 significant terms sorted by -log_10_(p-value) for shared KEGG pathways. Size of circle represents number of genes included in each term, while X-axis represents calculated Odds ratio. **c** Uniform manifold approximation and projection (UMAP) of 5 DMG patient tumour samples showing *FN1* expression. Each dots represents individual cells. *FN1* differential expression in microglia versus all other cell types is included in the panel (**d**), Immunoblot analysis and quantification of FN1 protein expression in iPSC microglia after 6- and 24-hour exposure with SF8628 DMG cells. **e** Quantification of the SF8628 DMG cell invasion capability in presence of iPSC microglia and FN1 Inhibitors RGDS and Avapritinib. Data in (**a**, **b**) originates from mutual comparison of 3 independent in vitro biological replicates of microglia exposed to SF8628 and 5 human DMG biopsies. Data in (**c**) originates from 5 DMG patient tumour samples. Human iPSC-related data are represented as mean ± SEM from 3 (**d**, **e**) independent biological replicates. Statistical annotations *p < 0.05; **p < 0.01; for indicated comparisons. See Suppl. Fig. 5, for similar analysis on a 3-hour time point.

Enrichment analysis for GO biological process terms using the overlapping DEGs found to be common between TAMs isolated from DMG biopsies and microglia exposed for 3 h to SF8628 DMG cells identified *FN1, COL1A1, COL1A2, COL4A2, COL5A2*, and *COL6A1* as candidate genes whose expression is associated with biological processes such as extracellular matrix organisation, regulation of cell-substrate adhesion, glial cell fate determination, cell-matrix adhesion, and plasma membrane regulation (Suppl. Fig. 5a). Corresponding KEGG pathway analysis confirmed a strong link for these shared DEGs to ECM remodelling (Suppl. Fig. 5b). When microglia exposed for 6 hours to SF8628 DMG cells were used for the comparison with TAMs isolated from DMG biopsies, overlapping DEGs included *FN1, COL1A1, COL1A2, COL5A2*, and *ITGA3*, with an enrichment in GO biological process terms for ossification, regulation of Wnt signalling, collagen fibril organisation, response to steroid hormones, and cell-matrix adhesion (Fig. 5a). KEGG pathway analysis at this time point revealed similar ECM-related alterations to those observed at the 3-hour time point (Fig. 5b). Hence, these human transcriptomic data further support a role for microglia (alternatively, more broadly TAMs) in driving a remodelling of the extracellular matrix, including the production of FN1, within the DMG tumour microenvironment.

### Microglia are the primary *FN1*-expressing cells in human DMG H3K27M tumours

Multiple cellular components of the tumour microenvironment can contribute to extracellular matrix assembly, and microglia may not be the only source of FN1. To clarify which cell populations express FN1 in human DMG, we re-analysed a publicly available single-cell RNA-seq dataset generated from 19 pHGG, including five DMG H3K27M cases [41].

Restricting the analysis to the five DMG H3K27M cases revealed that *FN1* expression is predominantly localized to the myeloid compartment, with microglia representing the statistically major *FN1*-expressing cell population within DMG tumours. In contrast, all other investigated cell types showed minimal or negligible *FN1* transcript levels (Fig. 5c). When extending the analysis to the full cohort of 19 pHGG samples, the myeloid compartment remained the dominant source of *FN1*-producing cells (Suppl. Fig. 5d).

To strengthen the human relevance of these findings, we conducted additional experiments using human induced pluripotent stem cell (iPSC)–derived microglia, an emerging tool to study microglial functions in health and disease [42]. Following exposure to SF8628 DMG cells, iPSC-derived microglia exhibited a marked increase in FN1 protein expression compared with both untreated iPSC-derived microglia and those stimulated with SF188 pHGG cells, as shown by immunoblot analysis (Fig. 5d). Moreover, treatment of iPSC-derived microglia with the FN1 inhibitors RGDS or Avapritinib significantly reduced their intrinsic invasion capacity and diminished their ability to promote SF8628 DMG cell invasion in Matrigel-based transwell assays (Fig. 5e; Suppl. Fig. 5f).

Together, these findings support the conclusion that human microglia respond to DMG-derived cues by upregulating FN1 and that microglia constitute a principal source of FN1 within the DMG tumour microenvironment.

### Validation of ECM component expression in human DMG H3K27M tissue samples

**Fig. 6:**
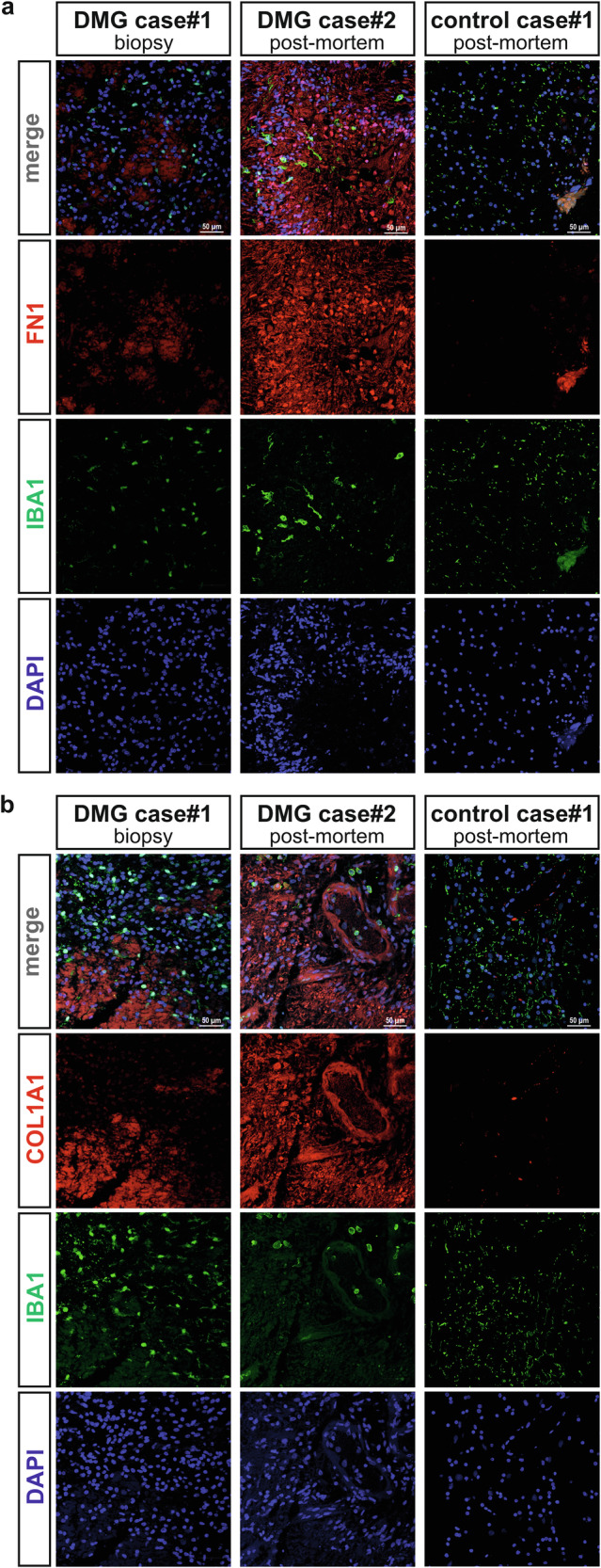
Human DMG H3K27M tumours exhibit increased FN1 and COL1A1 protein expression. **a** Confocal microscopy imaging of two human DMG tumours (one biopsy and one post-mortem samples), and one age-matched brainstem control case (post-mortem sample), with immunofluorescence staining for FN1 and IBA1. **b** Confocal microscopy imaging of the same cases as in panel a, with immunofluorescence staining for COL1A1 and IBA1. DAPI used as nuclear counterstain, scale bars(50 µm). Representative images originate from 2 independent DMG cases (1 biopsy, 1 post-mortem) with aged matched healthy subject (post-mortem). See Suppl. Fig. 6, for additional two human DMG tumours, and one age-matched brainstem control case.

To gain further validation that the expression of COL1A1 and FN1, two of the identified microglial ECM-related factors, are altered in the context of human DMG tumours, their protein expression was analysed by immunofluorescence in the same four DMG archival tissue samples obtained from the BRAIN UK biobank and used for the detection of microglia depicted in Fig. 1 and Suppl. Fig. 1. Compared to control cases, which exhibited no specific staining, DMG biopsies showed increased expression of both FN1 and COL1A1. As controls, tissue samples from two cases without neurological causes of death were included. IBA1 immunofluorescence co-staining was used to detect the microglial/TAM cell population within these archival tissue samples. Compared to control cases, which exhibited limited to no specific staining, DMG biopsies showed an increased expression for both FN1 and COL1A1. Notably, when comparing diagnostic biopsies to postmortem tissue, we observed a greater area of deposit coverage typical with high signal intensity (Fig. 6; Suppl. Fig. 6).

Collectively, the transcriptomic data from both microglia exposed to DMG cancer cells, TAMs isolated from human DMG biopsies, as well as the protein expression analysis for COL1A1 and FN1 in human archival DMG biobank tissues, support the concept of a microglia/TAMs-mediated remodelling of the ECM within the tumour microenvironment that could impact on the progression of DMG and potentially even hold prognostic value.

### Validation of ECM component expression and their prognostic value in DMG H3K27M across human cohorts

**Fig. 7:**
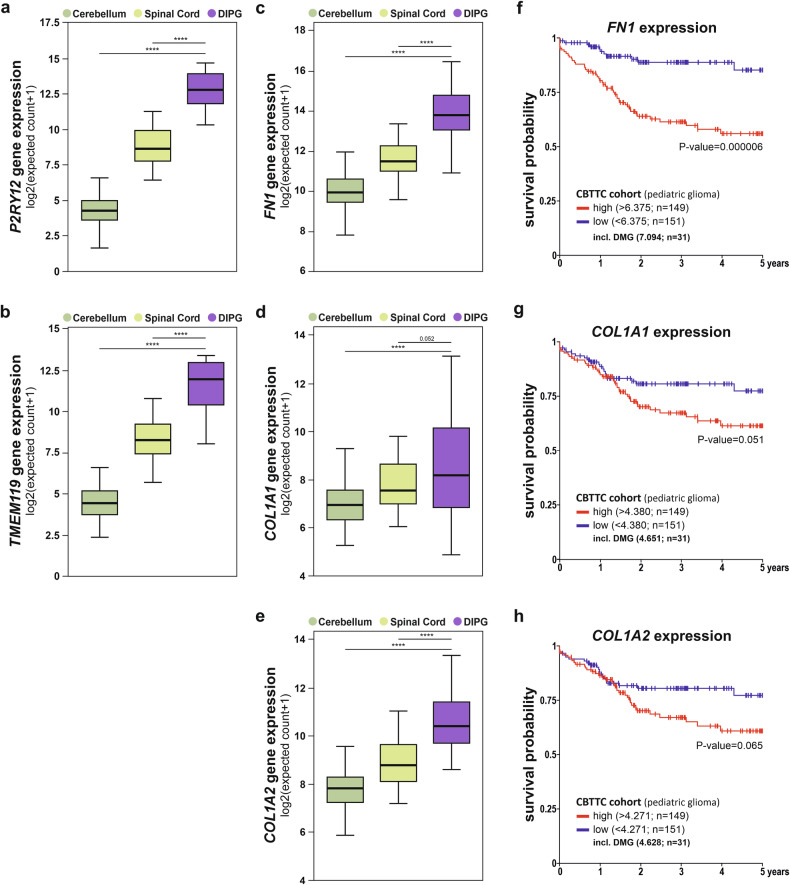
Validation of ECM component expression and prognostic value in DMG H3K27M human cohorts. **a**, **b** Gene expression levels for *P2RY12* (**a**), and *TMEM119* (**b**) in 16 DMG H3K27M subjects were extracted from the Gabriella Miller Kids First Pediatric Research Program (Kids First) cohort. The gene expression of these microglial markers in 60 spinal cord cervical C1 and 117 cerebellum brain region samples from healthy individuals, extracted from the Genotype-Tissue Expression Project (GTEx) cohort, were used for comparison. **c**–**e** Similar analyses as in panels a and b for *FN1* (**c**), *COL1A1* (**d**), and *COL1A2* (**e**) gene expressions. **f**, **g** Gene expression levels for *FN1* (**f**), *COL1A1* (**g**), *COL1A2* (**h**) and clinical data for a maximal 5-year period were extracted for 300 paediatric glioma subjects from the Children’s Brain Tumor Tissue Consortium (CBTTC) to assess their potential prognostic values. High and low expression were defined by the median value. Median expression for the gene of interest in 31 DMG, H3K27M subjects are indicated in the respective Kaplan-Meier 5-year survival curves. See Suppl. Fig. 7, for additional analyses performed on the Pacific Pediatric Neuro-Oncology Consortium (PNOC) and CBTTC human DMG cohorts.

Given the rarity of paediatric brain neoplasms and the challenges in obtaining precise control cases, we expanded our analysis to publicly available patient cohorts to further strengthen our findings. First, we utilised genomic and transcriptomic data for DMG H3K27M subjects from the Gabriella Miller Kids First Pediatric Research Program (referred as Kids First), initiative focused on identifying genetic contributors to childhood cancer [43]. and transcriptomic data from the Genotype-Tissue Expression (GTEx) Project, that provide a resource for healthy tissue reference [44]. to evaluate the expression of genes encoding microglial markers and ECM components in DMG H3K27M samples as compared to healthy tissue from anatomically relevant regions, including the spinal cord (Cervical C1) and cerebellum, which are adjacent to the midline (Suppl. Fig. 7a). In the DMG H3K27M subjects, a significant increase in the expression of *P2RY12* and *TMEM119*, microglial markers, was observed as compared to healthy controls (Fig. 7a, b) [45, 46]. The increased gene expression for these microglial markers in bulk RNA-seq from DMG H3K27M tumours, further confirmed the observation made with IBA1 immunofluorescence analysis on archival DMG H3K27M tissue samples, that microglial content is increased in these paediatric brain neoplasms (Fig. 1). In the DMG H3K27M subjects, a significant increase in the expression of *FN1, COL1A1* and *COL1A2* was also observed as compared to healthy controls (Fig. 7c–e). The increased gene expression for these ECM components in bulk RNA-seq from DMG H3K27M tumours further confirmed the observation made with FN1 and COL1A1 immunofluorescence analysis on archival DMG H3K27M tissue samples, that the DMG tumour microenvironment exhibits an increased expression for ECM components (Fig. 6; Suppl. Fig. 6).

To validate these findings across independent datasets, analyses were performed on two additional human cohorts from the Pacific Pediatric Neuro-Oncology Consortium (PNOC) and the Children’s Brain Tumor Tissue Consortium (CBTTC), open-access biorepositories with clinical and molecular data from paediatric brain tumours [47, 48]. Gene expression for *FN1*, *COL1A1*, and *COL1A2* were found to be elevated in brain neoplasms defined as DMG/DIPG H3K27M in PNOC cohort and DMG H3K27M in the CBTTC cohort at diagnosis, reported as initial tumours (Suppl. Fig. 7b, c). The CBTTC cohort also included data for progressive tumours, which showed similar expressions for *FN1*, *COL1A1*, and *COL1A2*, as compared to the initial tumours, suggesting that the increase in expression of these ECM components observed in DMG tumours could be an early event in the physiopathology of these paediatric brain neoplasms (Suppl. Fig. 7c).

To assess the potential clinical relevance of these ECM components, we conducted survival probability analysis in the CBTTC cohort, which includes longitudinal data for 300 paediatric glioma patients, including 31 DMG H3K27M subjects. This analysis demonstrated that an increased *FN1* expression was significantly associated with a worse 5-year survival (Fig. 7f), whereas increased *COL1A1* and *COL1A2* expressions exhibited a trend towards a negative correlation with prognosis, approaching statistical significance (Fig. 7g, h). In this human cohort, all DMG H3K27M cases exhibited high expression levels of *FN1*, *COL1A1* and *COL1A2*, as determined by the cohort’s median expression values, as shown in the corresponding panels. These clinical findings further support the role of ECM alterations in DMG progression and highlight FN1 as a potential prognostic marker for DMG H3K27M tumours.

## Discussion

DMG is a highly infiltrative neoplasm characterised by widespread and diffuse invasion into critical anatomic compartments within the midline regions. This diffuse invasion is a major obstacle for DMG treatment as it precludes surgical resection as a therapeutic option and contributes to the failure of current therapeutic modalities, such as focal radiotherapy [49]. One pre-requisite for neoplastic cells to infiltrate is adhesion to ECM components. The ECM is mainly composed of fibrous, insoluble, and high molecular weight proteins such as collagens, laminins, and fibronectin that provide physical and mechanical support for cells. Tumour cell adhesion to the ECM is governed by cell-surface ECM receptors, such as integrins [50]. The expression of laminin-associated integrins and fibronectin-associated integrins have been reported to be upregulated across a cohort of patient-derived DMG cell cultures including SU-DIPG-XXI, SU-DIPG-XXV, SU-DIPG-VI, and SU-DIPG-XIII-FL [51]. ECM components and their inhibition have been explored as potential therapeutic targets in adult-type diffuse glioma, such as glioblastoma [36, 52, 53]. However, the role of ECM remodelling in driving human DMG progression, as well as the cells within the tumour microenvironment that may contribute to ECM remodelling, remain poorly understood.

A meta-analysis of transcriptomic, genomic, and clinical data from three human DMG cohorts, i.e., Kids First, PNOC and CBTTC, revealed high gene expression for *FN1*, *COL1A1*, and *COL1A2* in the tumour mass of DMG H3K27M subjects. Analysis for FN1 and COL1A1 protein expression in archival biobank tissue confirmed elevated expression of these two ECM components, seen as deposits and tracks, in tumour mass from DMG H3K27M subjects, as compared to control aged, matched cases. This analyses, however, did not provide us with the cellular source responsible for producing these ECM components. However, bulk RNA-seq performed on microglia exposed to SF8628 DMG H3K27M cells (as compared to microglia alone, or microglia exposed to SF188 pHGG H3.wt cells), as well as bulk RNA-seq performed on tumour-associated myeloid cells isolated from human DMG H3K27M tissue samples, showed that microglia exhibit robust up-regulation of gene expression for several ECM components including *FN1, COL1A1*, and *COL1A2*. Genes encoding for laminin were not found to be significantly affected. Additional analyses performed for the expression of microglial markers, IBA1, *P2RY12* and *TMEM119*, in archival biobank DMG tissues and the Kids First human DMG cohort confirmed an increased presence of microglia in DMG tumours. Single-cell transcriptomic profiling of tumoral and non-tumoral cell populations within human DMG H3K27M tissue samples revealed that microglia constitute the predominant FN1-expressing cell type in these tumours. Although microglia emerge as the principal source of FN1 in DMG H3K27M tumours, it is important to acknowledge that tumour-cell–derived FN1 may also contribute to extracellular matrix remodelling in a patient- or subtype-dependent manner. Indeed, the single-cell RNA-seq dataset shows that a subset of tumour cells exhibits detectable *FN1* transcript levels. Supporting this possibility, the RGDS peptide, FN1 inhibitor, was found to reduce the invasive capacity of one of the three tested DMG cell lines, i.e., DIPG-XVII, suggesting that tumour-derived FN1 may have functional relevance in certain context. We hypothesize that the ECM components secreted by microglia could support DMG cancer cells invasion capacity. Exogenous FN1, as well as laminin, but not collagens, have been reported to support SU-DIPG-XIII-FL spheroid adhesion and migration in culture [51]. A screen of natural compounds for their ability to inhibit proliferation and anchorage-independent growth in SF8628 DMG H3K27M cells identified six candidates. Among the mechanisms underlying their effects, reduced FN1 expression was found to play a key role [54]. *FN1* is among the most upregulated genes in glioblastoma, and its expression inversely correlates with patients’ survival [55]. In this context, fibronectin has been shown to promote glioma stem cell proliferation, invasion, and therapeutic resistance [56]. However, although tumour-associated myeloid cells are known to contribute to extracellular matrix remodelling, their role as a direct source of fibronectin and the functional significance of this contribution to glioma invasion remain incompletely defined. Our findings extend this framework by identifying microglia as a major source of FN1 in DMG and demonstrating its functional role in promoting tumour cell invasion.

The RGD motif represents the minimal integrin-recognition sequence within FN1, and the RGDS peptide functions as an antagonist by blocking this site, thereby preventing FN1-mediated cell adhesion. Accordingly, RGDS has been explored as a potential therapeutic strategy for glioblastoma [36]. In a recent large-scale drug repurposing screen, Avapritinib was identified as a potent FN1 inhibitor that forms a highly stable complex with FN1 and induces structural alterations in its extra domain A (EDA) region [38]. Avapritinib has also been shown to cross the blood-brain barrier, reach therapeutic concentrations in the brain, and significantly reduce tumour growth in DMG mouse models [39]. Furthermore, a subset of paediatric and young adult patients with *PDGFRA*-altered high-grade gliomas exhibited clinical responses to Avapritinib in the same study, highlighting its translational relevance [39]. Chemical inhibition of microglial FN1 using RGDS or Avapritinib treatments, as well as genetic silencing of microglial *Fn1* using siRNAs, each resulted in reduced microglial invasiveness and diminished microglia-mediated enhancement of DMG cancer cell invasion.

Patient-derived 3D model studies have demonstrated that highly invasive DMG cells migration is characterised by reduced adhesion and increased actomyosin contractility, driven by an activation of MEK/ERK and Rho/ROCK signaling [57]. In this context, our data identify microglia as a major source of FN1 within the DMG niche, with robust upregulation observed in both experimental models and human tumours. FN1 may function as a signalling scaffold that modulates integrin-dependent pathways and reinforces ERK and Rho/ROCK signalling. We therefore propose that microglia-derived FN1 contributes to a permissive invasive niche by enhancing cytoskeletal contractility and facilitating plasticity between migratory states, rather than simply promoting adhesion. This model is supported by our functional data demonstrating that genetic or pharmacological inhibition of microglial FN1 significantly reduces DMG cell invasion, highlighting microglia-driven ECM remodelling as a critical and potentially targetable regulator of DMG dissemination.

Although, the upstream signals responsible for microglial activation remain to be identified TGF-β signalling represents a particularly plausible driver of microglial activation, given its well-established role in regulating extracellular matrix production, including fibronectin expression, across multiple cellular systems. In glioma, TGF-β has been directly linked to fibronectin expression and tumour progression through the induction of epithelial-to-mesenchymal transition (EMT)-associated programmes [58]. Beyond its role in tumour cells, TGF-β has been shown to directly reprogramme microglia towards an extracellular matrix-producing state, including the induction of fibronectin expression in other contexts such as the ageing brain [59]. Taken together, tumour-derived TGF-β may act as an upstream regulator of microglial FN1 expression in DMG. However, it is likely that TGF-β represents only one component of a broader network of tumour-derived signals that govern microglial activation. Additional pathways, including inflammatory cytokines, integrin signalling, extracellular vesicles, and metabolic cues, may act in parallel or in concert to shape the microglial transcriptional response.

Our findings support a model in which DMG cells actively reprogramme microglia to adopt an ECM-producing, pro-tumoral phenotype, with FN1 acting as a key effector of this interaction. Microglial FN1 secretion contributes to a permissive and signalling-active microenvironment that enhances tumour cell motility and invasion. Importantly, targeting FN1 through genetic or pharmacological approaches was sufficient to disrupt these microglia–tumour interactions and reduce invasion, highlighting its functional relevance. These results identify microglia-driven ECM remodelling as a critical and targetable component of DMG biology and suggest that disrupting microenvironmental support mechanisms may represent a complementary strategy to current tumour-directed therapies.

## Materials and methods

### Cell culture

BV-2 microglia (RRID: CVCL_0182; gift from Guy Brown, University of Cambridge), primary patient-derived SF188 pHGG cells (RRID: CVCL_6948) (gift from Stefan Pfister, German Cancer Research Center), primary patient-derived SF8628 DIPG cells (RRID: CVCL_IT46, MERCK, Cat. #SCC127), primary-patient derived SU-DIPG-XIII (RRID: CVCL_IT41), and SU-DIPG-XVII (RRID: CVCL_C1MW) (gift from Michelle Monje, Stanford University) have been used in this study. BV-2, SF188, and SF8628 cells were cultured in high glucose Dulbecco’s modified Eagle’s medium (DMEM) supplemented with penicillin (100 μg/ml), streptomycin (100 μg/ml) and 10% foetal bovine serum (FBS) (all from Gibco™ ThermoFisher Scientific) at 37°C and 5% CO2. SU-DIPG-XIII and SU-DIPG-XVII cells were maintained as neurospheres in tumour stem media (TSM) that contained DMEM/F12, Neurobasal media, B27 supplement without vitamin A (all from Invitrogen), human bFGF (20 ng/ml), human EGF (20 ng/ml), human PDGF-AA (10 ng/ml), human PDGF-BB (10 ng/ml) (all from Shenandoah Biotech) and heparin (2 ug/ml) (STEMCELL technologies). All cell lines were regularly checked for mycoplasma with Venor^™^GeM mycoplasma detection kit (Minerva Biolabs).

### Generation of human iPSCs from skin fibroblasts

Ethical permission for reprogramming human cells (Dnr. 2012/208-31/3 with addendum 2012/856-32 and 2015/1097-31/1) have been approved by the Ethical review board (Regionala etikprövningsnämnden i Stockholm). Human iPSC cells were taken from the iPS core facility at Karolinska Institutet, as previously described [60]. Briefly, iPSCs were generated from skin fibroblasts of a healthy individual [61]. CytoTune™-iPS reprogramming kit (Invitrogen) was used to reprogram these fibroblasts to iPSCs, which were picked manually for the first passage and passaged enzymatically later as single cells. Thus, the generated iPS cell lines were characterized by applying different approaches, including karyotyping and genome-wide transcriptional assay (Illumina HT 12 v.4) followed by bioinformatical assay (PluriTest) [62].

### Microglial derivation from iPSCs

Microglia-like cells were generated from Control iPSCs as described earlier [42]. Briefly, the iPSC colonies were grown in an essential 8 (E8) medium. When these cells reached about 80% confluence, they were dissociated as single cells using TrypLE Select (Invitrogen). Thereafter, these cells were seeded on matrigel-coated plates at a density of 50.000 cells/cm^2^ in E8 medium containing activin A (R&D 338-AC; 7.5 ng/ml), BMP4 (R&D; 30 ng/ml), CHIR 99021 (Sigma, 3 μM) and ROCK inhibitor (Revita, Thermo Fisher scientific). After 18 h, the E8 medium was replaced with essential 6 (E6) medium and added activin A (10 ng/ml), BMP4 (40 ng/ml), and IWP2 (Selleck; 2 μM). The next day, the medium was changed to E6 medium containing activin A (10 ng/ml), BMP4 (40 ng/ml), IWP2 (2 μM), and bFGF (R&D; 20 ng/ml). The following day, cells were dissociated and plated 50,000 cells per cm^2^ in E6 medium containing vascular endothelial growth factor (VEGF; R&D) (15 ng/ml), bFGF (5 ng/ml), and Revita (1:100). After 24 h, the E6 medium was replaced with fresh E6 medium containing VEGF (15 ng/ml) and bFGF (5 ng/ml). After 2 days, cells were fed with E6 medium containing VEGF (15 ng/ml), bFGF (5 ng/ml), SCF (200 ng/ml), and IL6 (20 ng/ml). Thereafter, the medium was changed to E6 medium with SCF (100 ng/ml), IL6 (10 ng/ml), TPO (30 ng/ml) and IL3 (30 ng/ml) every other day. Then, microglial precursors were collected as suspension, which were cultured again in 75% IMDM, 25% DMEM F12 medium containing B-27 supplement, GLUTAMAX (1.5 mM), and IL34 (100 ng/ml) and M − CSF (20 ng/ml) for 10 days before co-culture.

### Segregated co-culture experiments

For RNA sequencing and validation experiments on BV-2 microglia segregated co-cultures were performed whereby 2×10^6^ SF8628 DMG cells or SF188 pHGG cells were plated in 10 cm culture dishes (Sarstedt) and 120,000 BV-2 microglia cells were plated on coverslip. Cells were plated in complete DMEM medium containing 10% FBS overnight. The next day the media was removed from the SF8628 DMG/SF188 pHGG cell culture dishes and replaced with DMEM containing 5% FBS, 4 coverslips containing BV2-microglia were transferred to each 10 cm dish and left for 3, 6, 24, or 48 h.

For validation experiment on iPSC-microglia segregated co-cultures were performed whereby 600,000 SF8628 DMG cells or SF188 pHGG cells were plated in 6 well plate (Corning) and 120,000 iPSC-microglia cells were plated on previously coated coverslip. Cells were plated in complete DMEM medium containing 10% FBS overnight. The next day the media was removed from the SF8628 DMG/SF188 pHGG cell culture plates and replaced with DMEM containing 5% FBS, 1 coverslip containing iPSC-microglia was transferred to each well and left for 6 or 24 h.

### Gene silencing by transfection of small interfering RNAs pools

For transient gene expression silencing by siRNAs, non-targeting control and *Fn1* ON-TARGET plus SMARTpools siRNAs, were obtained from Dharmacon. Transfection of BV-2 cells was carried out with Lipofectamine 3000 (Invitrogen). ***Fn1*** (mouse, NM_0011276408) **ON-TARGET plus SMARTpools siRNA** (Dharmacon, L-043446): AGAACAAACACUAACGUAA; GGUCAUUUCAGAUGCGAUU; GGAGAGAGAUGCACCGAUU; GGUUCAGACUCGAGGCGGA. **Non-targeting siRNA pool** (si-Control; Dharmacon, D-001810): UGGUUUACAUGUCGACUAA; UGGUUUACAUGUUGUGUGA ; UGGUUUACAUGUUUUCUGA ; UGGUUUACAUGUUUUCCUA.

### Invasion assays

SF8628 DMG cells were labelled with 2,5 μM of Cell Tracker (C70255, Invitrogen) in DMEM medium containing 10% FBS for 30 minutes at 37 °C. A mixture containing Cell Tracker labelled SF8628 cells (15,000), BV-2 microglia or iPSC microglia (7,500), 1,6 mg/ml Matrigel (Corning 354234), and either 10 μM RGDS peptide (Abcam, ab230365), 0,5 μM Avapritinib (Fisher, BLU-285) or DMSO vehicle control was prepared and added to the upper chamber of 8 μm pore-size PET membrane insert (Transwell, Corning). The lower chamber contained 1 ml of DMEM medium containing 10% FBS to establish a chemoattractant gradient. After 24 h of incubation at 37 °C, non-invading cells were gently removed from the upper surface of the insert membranes using cotton swabs. Inserts were fixed with 4% paraformaldehyde and mounted. Cell Tracker-positive (SF8628 DMG cells) and Cell Tracker- negative (BV-2 microglia or iPSC microglia) migrated cells were counted under fluorescent microscopy (Axio Observer 3, ZEISS) in 2 inserts and 4 fields per insert per condition using ImageJ cell counter.

SU-DIPG-XIII and SU-DIPG-XVII (DMG H3K27M) cells were first labelled with 2 μM carboxyfluorescein succinimidyl ester (CFSE; Invitrogen C34554) in PBS for 20 min at 37 °C. Excess of CFSE dye was removed by washing the cells in DMEM medium containing 10% FBS. A mixture containing CFSE-labelled DMG cells (150,000), BV-2 microglia (50,000 cells), Matrigel (Corning, diluted 1:5 in DMEM medium containing 5% FBS), and either 10 μM RGDS peptide (fibronectin inhibitor; Abcam, ab230365) or DMSO vehicle control was prepared and added to the upper chamber of 8 μm pore-size PET membrane inserts (Transwell, Corning). The lower chamber contained 1 ml of DMEM medium containing 10% FBS to establish a chemoattractant gradient. After 24 h incubation at 37 °C, non-invading cells were gently removed from the upper surface of the insert membranes using cotton swabs. Inserts were fixed with 4% paraformaldehyde and mounted. CFSE-positive migrated cells were counted under fluorescent microscopy (Axio Observer 3, ZEISS) in 2 inserts and 4 fields per insert per condition using ImageJ cell counter.

### Immunobloting

Total protein extracts were made directly in Laemmli buffer by scraping of the cells. Samples were sonicated (Diagenode, Bioruptor Pico) and boiled. Proteins were then separated in 10% SDS-polyacrylamide gel for COL1A1 and 4-15% Mini-Protean tgx gels or alternatively in 3–8% Criterion XT Tris-Acetate precast gels for FN1 by electrophoresis and blotted onto 0.45 μm pore-size nitrocellulose membranes using the Mini Trans-Blot or Criterion wet transfer system (all from BioRad). Membranes were blocked in 3% BSA in PBS and incubated overnight at 4 °C with anti-Fibronectin primary antibody (1:1000; Abcam, ab2413) or anti-COL1A1 primary antibody (1:1000; Invitrogen, PA5-29569) in 3% BSA in 0.1% Tween 20 (Sigma-Aldrich) in PBS. Membranes were incubated with RDye® secondary antibodies (LI-COR Biosciences) according to the manufacturer’s instructions. Protein bands were visualized using the Odyssey CLx infra-red imaging system (LI-COR Biosciences) equipped with the software Image Studio Lite, version 5.2 (LI-COR Biosciences). All targeted proteins of interest were normalized to the selected housekeeping protein anti-actin (Sigma-Aldrich, A3853), and intensity of the bands was quantified using ImageJ software.

### RNA isolation, cDNA synthesis, and qPCR

Total RNA was isolated from cells using the RNeasy Plus Mini extraction kit (Qiagen) following the manufacturer’s instructions. cDNA was synthesized from 1 μg of mRNA using Oligo dT, dNTPs and Superscript IV (all Invitrogen). RT-qPCR was performed using SSoAdvanced Universal SYBR Green Supermix (Bio Rad) and run on CFX Duet Real Time PCR system (Bio Rad). *Actb* was used as a housekeeping gene for normalization. Results were calculated using delta Ct method and represented as a fold over control.

Sequences of primers used in this study (Forward, Reverse):

*Fn1 (mouse)*5´-TACAACAACCGGAATTACC-3´5´-GATACATGACCCCTTCATTG-3´

*Col1a1 (mouse)* 5´-CCGATGGATTCCCGTTCGAG-3´5´-GAGGCCTCGGTGGACATTAG-3´

*Actb (mouse)*5´-GATGTATGAAGGCTTTGGTC-3´ 5´-TGTGCACTTTTATTGGTCTC-3´

### Library preparation and RNA-sequencing

Total RNA was subjected to quality control with Agilent Tapestation according to the manufacturer’s instructions. Two hundred nanograms of total RNA was subjected to Illumina sequencing and libraries were prepared with the Illumina TruSeq Stranded mRNA kit which includes cDNA synthesis, ligation of adaptors, and amplification of indexed libraries. The yield and quality of the amplified libraries was analysed using Qubit by Thermo Fisher and the Agilent Tapestation. The indexed cDNA libraries were normalized and combined, and the pools were sequenced on the Illumina Hiseq 2000 generating 50 bp single-end reads.

### RNA-seq data and computational analysis

Basecalling and demultiplexing were performed using Illumina bcl2fastq v2.20.0.422 software with default settings, generating FASTQ files for further downstream mapping and analysis. STAR v2.7.5b was used to index the mouse reference genome (mm10/ GRCm38) and align the resulting fastq files. Mapped reads were then counted in annotated exons using featureCounts v1.5.1. The annotations and reference genome were obtained from Ensembl. The count table from featureCounts was imported into R/Bioconductor, and differential gene expression was performed using the EdgeR v3.30.3 package and its general linear model pipeline. For the gene expression analysis genes that had 1 count per million in 3 or more samples were used and normalized using TMM normalization.

*Volcano plot* - For the “positive versus negative” comparison, representing all differentially expressed genes (DEGs) for the following comparisons: (i) BV-2 microglia cocultured with SF188 pHGG cells versus monocultured BV-2 microglia, (ii) BV-2 microglia cocultured with SF8628 DMG cells versus monocultured BV-2 microglia, and (iii) BV-2 microglia from SF8628 DMG cells coculture versus BV-2 microglia from SF188 pHGG cells coculture, at both 3-hour and 6-hour time points, were created using the VolcaNoseR web app (https://huygens.science.uva.nl/), which displayed significance and fold change for the dataset together with gene symbols for the top 20 most highly regulated genes [63].

*Heatmap* - heatmaps depicting log2 fold change (log2FC) in gene expression between BV-2 microglia cocultured with SF188 pHGG cells or SF8628 DMG cells were generated using the Morpheus software from the Broad Institute (https://software.broadinstitute.org/morpheus/).

The following analyses were conducted on significant DEGs (*p*-value < 0.05) from microglia exposed to tumour cells compared to monoculture after exclusion of pseudogenes and non-transcripts. Gene ontology (GO) enrichment analysis for biological processes (BP) was performed using Metascape (v3.5.20240901)(https://metascape.org) [64]. Pathway enrichment analysis, including KEGG pathway and TRRUST transcription factor analysis, was performed using EnrichR [65]. Visual representations of bioinformatic analyses, including GO BP, KEGG, transcription factor enrichment, and heatmaps representing the expression of normalized genes across experimental conditions were generated using SRplot (https://www.bioinformatics.com.cn/srplot) [66].

To assess the potential relevance of DEGs from BV-2 microglia cocultured with SF8628 DMG cells, these genes were compared with a published dataset of genes from human tumour-associated macrophages/microglia (TAMs) from DIPG biopsies [24]. The normalized RNA-Seq dataset from the referenced study was kindly provided by the authors, and fold changes were calculated between DMG and control tissue samples in Excel. Genes exhibiting a log₂ fold change greater than 2 (log₂FC < −2 or > 2) were considered for the comparison. The overlapping genes between the two datasets were subsequently subjected to GO BP and KEGG pathway enrichment analyses, as described above.

### Patient sample single-cell RNA-Seq analysis

A single-cell RNA-Seq (scRNA-Seq) dataset was prepared from disaggregated cells obtained from 19 patient tumour samples of pHGG as previously described [41]. This dataset is accessible via the ALSF Pediatric Neuro-Oncology Cell Atlas (https://www.pneuroonccellatlas.org/), UCSC Cell Browser and Gene Expression Omnibus (GEO) with accession number: GSE126025. Low-expressing cell were removed from the pHGG dataset and then DMG samples were extracted (*n* = 5) into a separate object. Differential gene expression for *FN1* in the DMG and PHGG samples was performed using FindAllMarkers in Seurat (5.3.1) running under R(4.5.0) and FeaturePlot in Seurat was used to display gene expression [67, 68].

### Immunofluorescence analysis on BV-2 microglia

BV-2 microglia were grown on 12 μm coverslip and 24 h segregated coculture was performed with SF188 pHGG cells or SF8628 DMG cells. 4% paraformaldehyde-fixed cells were permeabilized in 0.3% PBS-T for 20-minutes and blocked in 10% goat serum for 1-hour at room temperature. Followed by 0.3% PBS-T washes and incubation with the indicated primary antibody rabbit anti-Col1a1 (1:250, ThermoFisher; PA5-29569), and rabbit anti-Fibronectin (1:500; Abcam, ab2413) at 4 °C overnight. Cells were washed three times with PBS 1X for 5 min before 1-hour incubation with secondary antibody goat anti-rabbit Alexa 488 (1:500; Fisher Scientific, 17177061), diluted in 0.3% PBS-T, at room temperature. Actin cytoskeleton was counterstained for 30 min with Phalloidin (1:400; Fisher Scientific, 10751144) Nuclei were counterstained for 10 min with Hoechst 34580 (1:500; Invitrogen). Samples were mounted with Fluoromount-G^®^ (Southern Biotechnology/AH Diagnostic, 0100-01). Images were taken under a fluorescent microscope (Zeiss LSM800-Airy). ImageJ/Fiji was used to analyse the fluorescence intensity of the images. These experiments were performed in 3 biological replicates.

### Immunofluorescence analysis on biobank human tissues

Experiments with human tissue were carried out with ethical approval from the local Ethical review board (Regionala etikprövningsnämnden i Stockholm; dnr 2021-06665-01) with paraffined tissues obtained from BRAIN UK at the University of Southhampton operating under strict ethical guidelines regarding informed consent. Sections were deparaffinized at 55 °C for 10-minutes and rehydrated in Xylene followed by decreasing concentrations of ethanol (100%, 90%, 80%, 70%). Heat induced antigen retrieval (Target Retrieval Solution, Dako) was performed using the 2100 Retriever (Diagnostic technology). Sections were washed in PBS and permeabilized in PBS-Triton 0.3% followed by 1 h incubation in 10% Normal Goat Serum. Sections were incubated with the indicated primary antibody mouse anti-human IBA1 (1:500, Synaptic Systems, 234011), rabbit anti-human COL1A (1:250, ThermoFisher, PA5-29569), and rabbit anti-human Fibronectin (1:500, Abcam, ab2413) diluted in blocking solution overnight at 4 °C. Sections were washed and incubated in secondary antibody mix (goat anti-mouse Alexa Fluor 488 (1:200; ThermoFisher Scientific, A-11029) and goat anti-rabbit Alexa Fluor 594 (1:200, ThermoFisher Scientific, A-11037)) for 1 h. Sections were washed in PBS and nuclei were counterstained for 10 min with Hoechst 34580 (1:500; Invitrogen). Samples were mounted with Fluoromount-G™ (Southern Biotechnology/AH Diagnostic, 0100-01). Pictures were taken under a confocal fluorescent microscope (Zeiss LSM800-Airy).

### IMARIS

Quantitative image analysis was performed using IMARIS software. The *Spots* detection algorithm was used to identify IBA1-positive cells based on size, shape, and fluorescence intensity. Spot diameter was estimated based on cell body size ( ~ 8–12 µm), and automatic thresholding was applied to isolate true positive signals. Background subtraction and signal smoothing were used to enhance spot accuracy. For each field, the total number of IBA1-positive cells per mm² was calculated, and fluorescence intensity values per spot (cell) were extracted using the Mean Intensity measurement for the red (IBA1) channel. These intensity values reflect relative IBA1 expression per microglial cell.

### Human cohorts’ analysis

*Gene expression analysis* – Genomic data (H3K27M mutational status), gene expression data (*P2RY12*, *TMEM119*, *FN1*, *COL1A1* and *COL1A2*) and clinical data from the Gabriella Miller Kids First Pediatric Research Program (Kids First; 16 DMG H3K27M cases), the Pacific Pediatric Neuro-Oncology Consortium (PNOC; 27 DMG H3K27M cases) and the Children’s Brain Tumor Tissue Consortium (CBTTC; 17 DMG H3K27M cases) cohorts were obtained using UCSCXenaTools or the Open Pediatric Brain Tumor Atlas (OpenPBTA) [47, 48]. Gene expression data from human healthy tissues (spinal cord cervical C1, and cerebellum) from the Genotype-Tissue Expression (GTEx) project, was used as controls [44].

*Survival analysis* - Gene expression data and clinical data for 300 paediatric glioma cases (including 31 DMG H3K2M cases) from the CBTTC cohort, were acquired using UCSCXenaTools [69]. Patient survival was analysed by Kaplan–Meier method. High and low gene expression groups were defined by median value and visualized employing survminer. Differences between the two groups were assessed for significance through log-rank test.

### Statistical analysis

All values are a mean of at least 3 independent biological replicates ± SEM. Statistical analysis was performed using GraphPad Prism (GraphPad Software, Version 10.0), the threshold for statistical significance was considered when the *p* value was less than 0.05. All the statistical analysis can be found in Suppl. table 3.

## Supplementary information

Supplementary Figures 1 to 7

Supplementary Table 1

Supplementary Table 2

Supplementary Table 3

Original Data

## Data Availability

The transcriptome dataset comparing BV-2 microglia exposed to SF8628 DMG cells or SF188 pHGG cells is available at the Gene Expression Omnibus with accession number: GSE309866. The bulk RNA-seq and scRNA-seq from human DMG biopsies are already published [24, 41]. All other data are available from the corresponding author upon reasonable request.
