# Supplementary material for: Microglia in diffuse midline glioma contribute to extracellular matrix remodelling and cancer cell invasion
Source: Cell Death Dis. 2026 May 30;17(1):517. doi: 10.1038/s41419-026-08891-y (PMC13222350; doi:10.1038/s41419-026-08891-y)
Supplement: Supplementary file 1 — Supplementary Figures 1 to 7 [file 41419_2026_8891_MOESM1_ESM.pdf]

Microglia in diffuse midline glioma contribute to extracellular matrix remodelling and cancer cell invasion.

Supplementary Figures

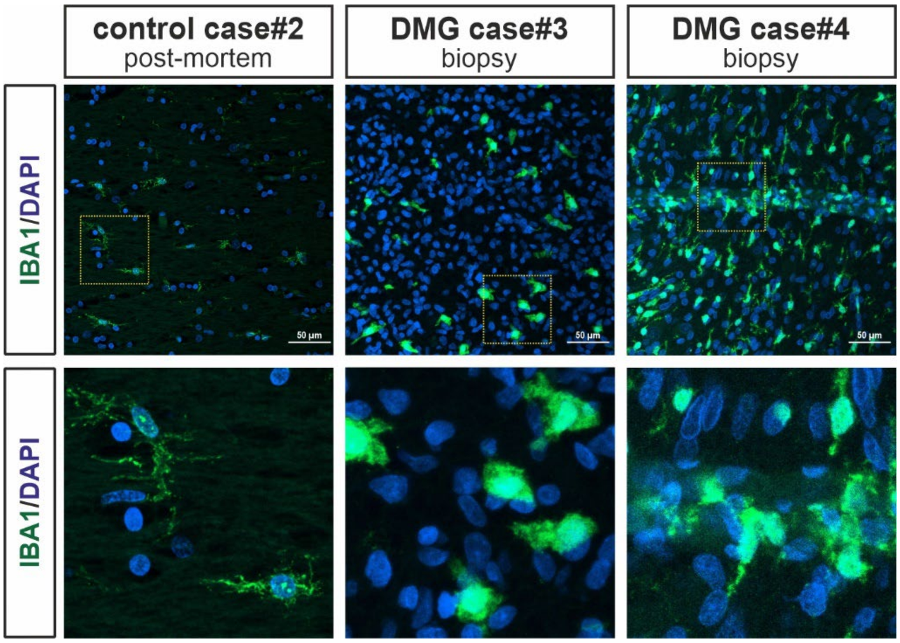

Supplementary Figure 1 | Increased presence of reactive microglia in human H3K27M DMG tumours.

Confocal microscopy imaging of two human DMG tumours (biopsy samples), and one age-matched brainstem control case (post-mortem sample), with immunofluorescence staining for IBA1. DAPI used as nuclear counterstain, scale bars (50 µm).

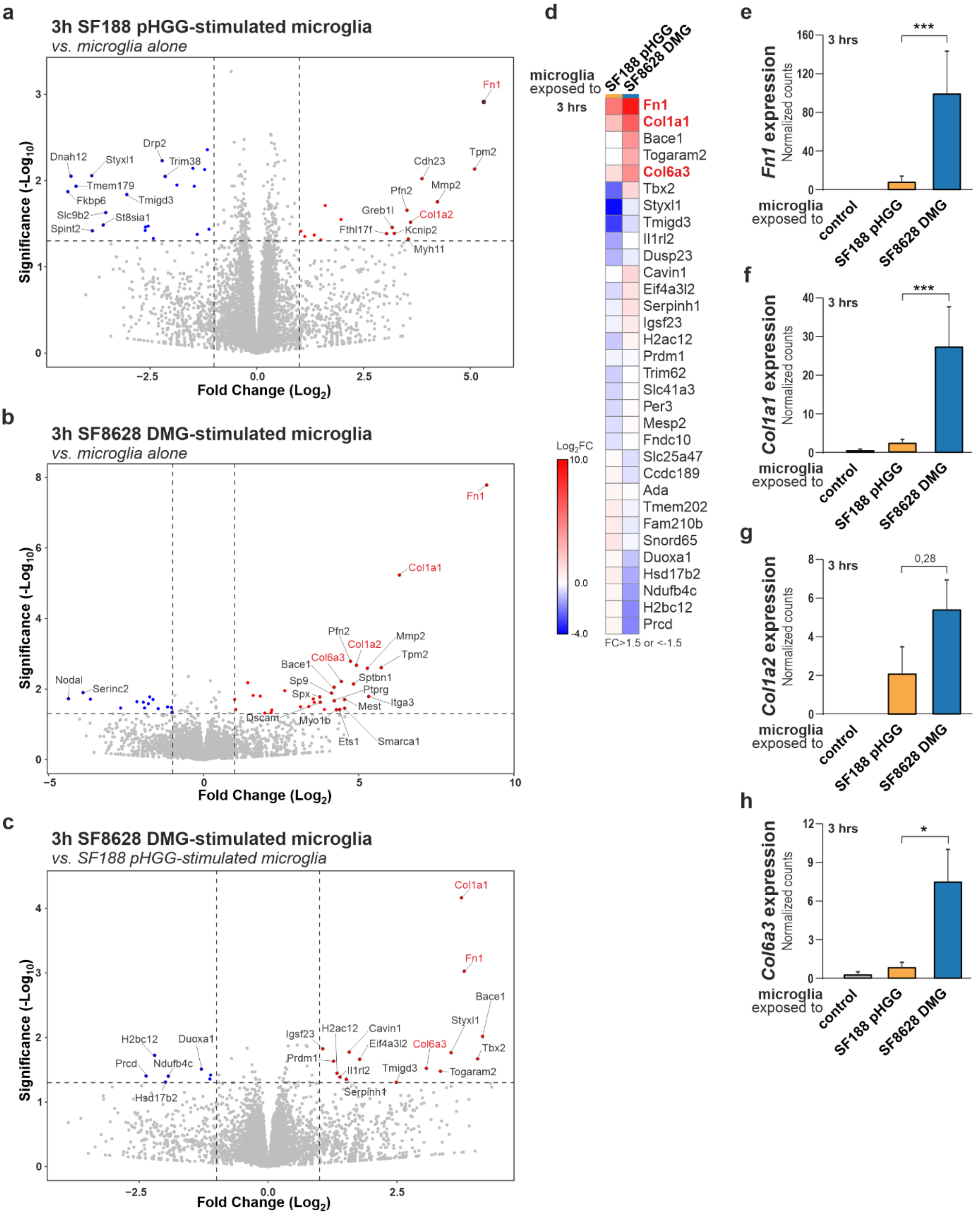

Supplementary Figure 2 | Microglia exposed to DMG H3K27M cells and pHGG cells exhibit different transcriptomic responses.

a,**b**, Volcano plots illustrating differentially expressed genes (DEGs) based on the $\log_2$(fold change) related to negative $\log_{10}$(p-value) between microglia exposed to SF188 pHGG cells for 3 hours and microglia alone (a) or between microglia exposed to SF8628 DMG cells for

3 hours and microglia alone (b).**c**, Volcano plots illustrating DEGs between 3 hours SF8628 DMG-stimulated microglia compared to microglia alone and 3 hours SF188 pHGG-stimulated microglia compared to microglia alone. Blue dots represent significantly downregulated genes with $\log_2$(FC) of maximally -1, and red dots significantly upregulated genes with $\log_2$(FC) at least 1. Names of the top 20 genes for up- or downregulated genes are depicted. **d**, Heatmap representation of genes found to be statistically differentially expressed (FC>1.5) between conditions described in panel c. Expression data for these genes were extracted from the comparisons of microglia exposed for 3 hours to SF188 pHGG cells compared to microglia alone or SF8628 DMG cells compared to microglia alone. **e-h**, *Fn1*, *Col1a1*, *Col1a2* and *Col6a3* gene expressions as normalized counts presented as mean ± SEM, in microglia exposed to SF188 pHGG cells for 3 hours, microglia exposed to SF8628 DMG cells for 3 hours and microglia alone. Data depicted in this figure originate from RNA-seq analysis of 3 independent biological replicates for each group. Statistical annotation * p<0,05; ** p< 0,01; and *** p< 0,001; for indicated comparisons.

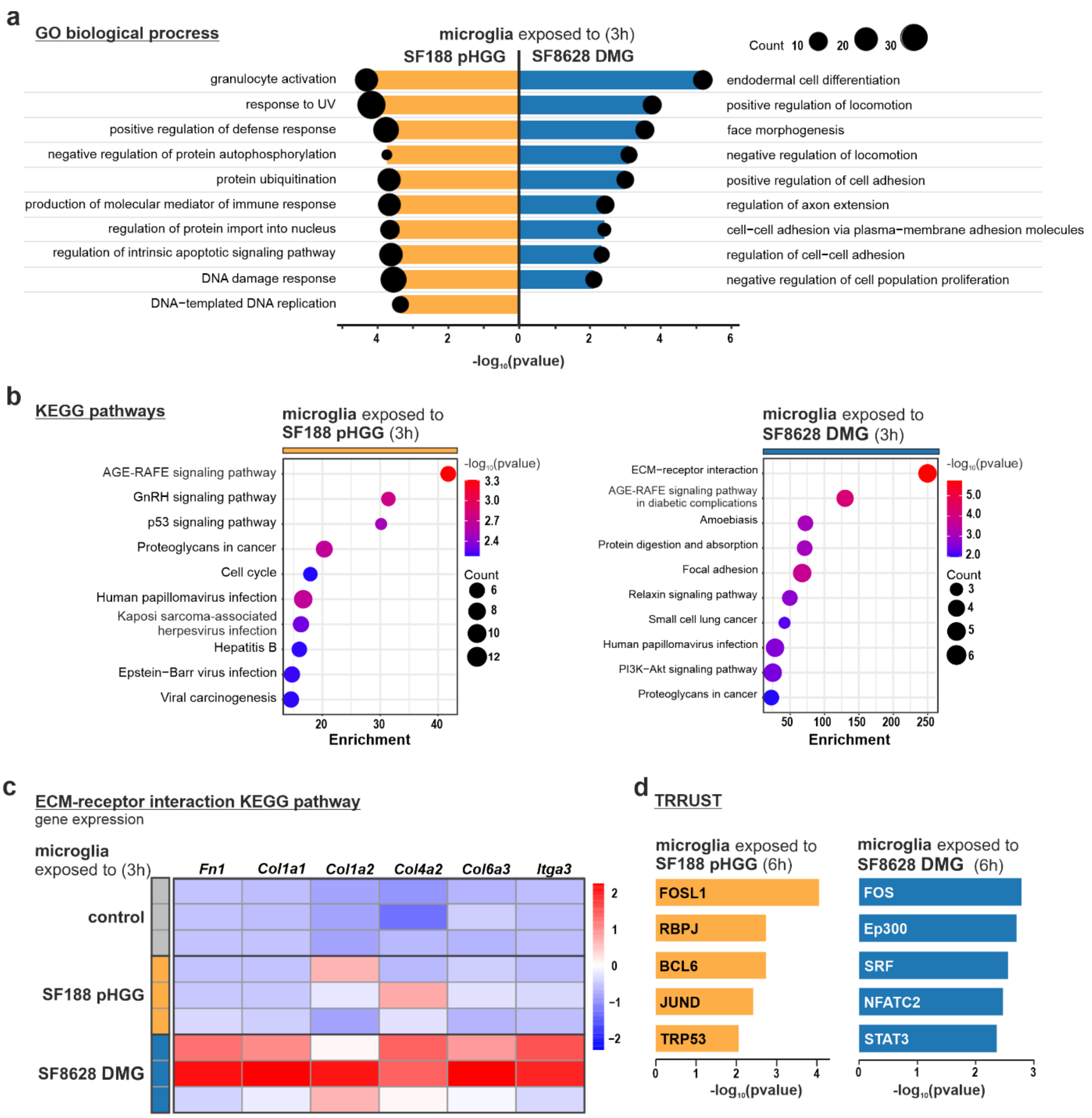

Supplementary Figure 3 | Microglia exposed to DMG H3K27M cells exhibit a transcriptomic response associated with extracellular matrix remodelling.

a, Analysis of enriched Gene Ontology (GO) terms for biological processes showing the top 10 significant terms sorted by -log_{10}(p-value) for both, microglia exposed to SF188 pHGG cells and SF8628 DMG cells at the 3-hour time point. Size of circle represent number of genes included in each GO BP term. **b**, KEGG pathways enrichment analysis that allows identification of significantly affected pathways related to upregulated genes in SF188 pHGG cells-stimulated microglia and SF8628 DMG cells-stimulated microglia. The top 10

significant terms sorted by -log_{10}(p-value) are displayed. Size of circles show number of genes included in each term and X-axis represent calculated enrichment. **c**, Heatmap represent genes included in ECM-receptor interaction KEGG pathway displaying expression of genes of interest between experimental conditions. **d**, TRRUST analysis, which allows the identification of potentially involved transcription factors based on the expression of their target genes in SF188 pHGG cells-stimulated microglia and SF8628 DMG cells-stimulated microglia. The top 5 genes sorted by -log_{10}(p-value) are shown. Data depicted in this figure originate from RNA-seq analysis of 3 independent biological replicates for each group.

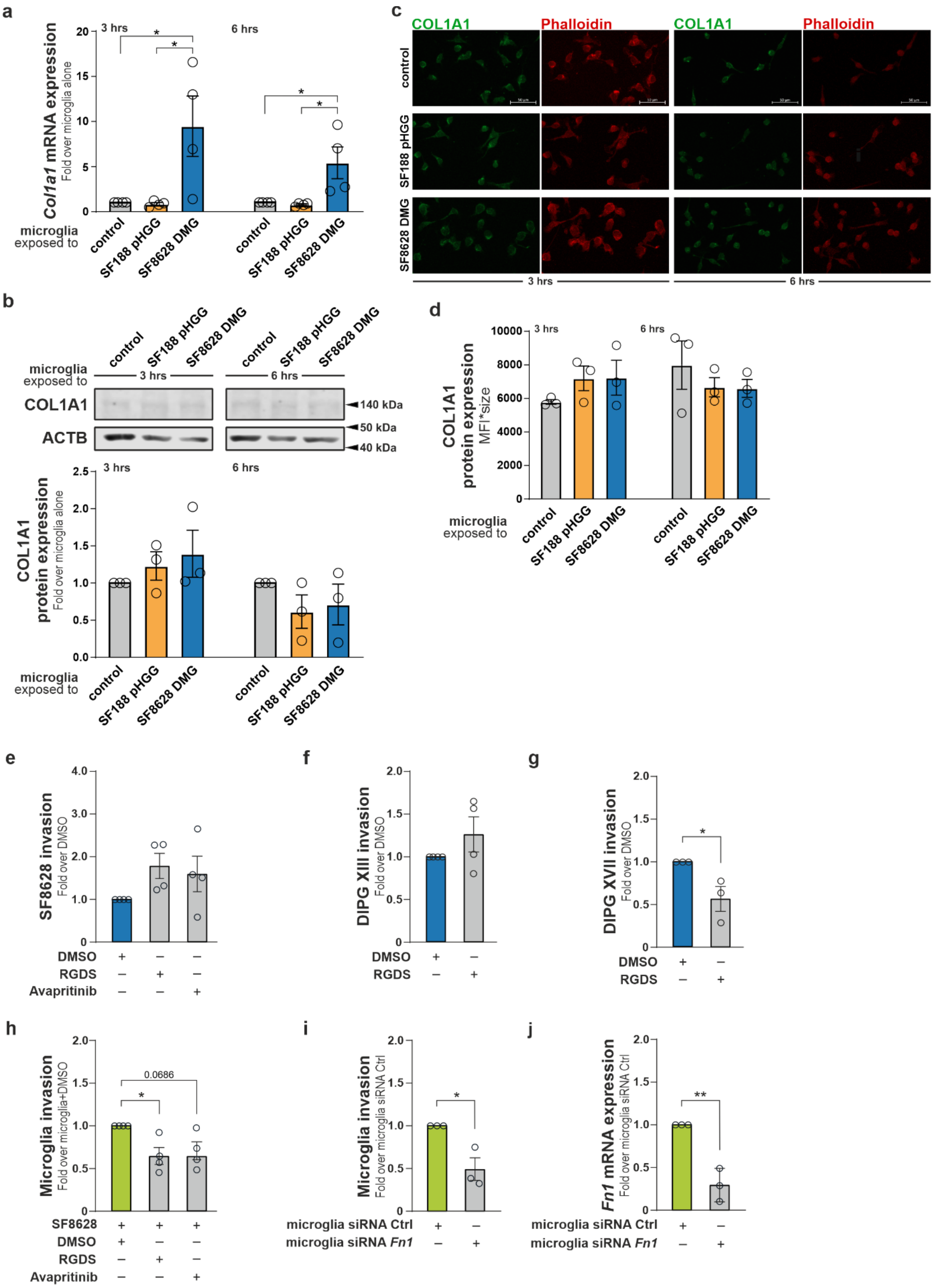

53

54 **Supplementary Figure 4 | Inhibition of fibronectin 1 derived from microglia**
55 **reduces DMG H3K27M cell invasiveness.**

a, RT-qPCR analysis confirms upregulation of *Col1a1* mRNA expression in microglia exposed to SF8628 DMG cells, as compared to microglia exposed to SF188 pHGG cells or microglia alone at 3- and 6-hour time point. **b,** Immunoblot analysis and quantification of COL1A1 expression in microglia exposed to SF188 pHGG cells or SF8628 DMG cells for 3 and 6 hours. **c,d** Immunocytochemistry analysis of COL1A1 in BV-2 microglia exposed to SF188 pHGG cells or SF8628 DMG cells for 3 and 6 hours. Phalloidin was used as counterstaining for F-actin. Quantification COL1A1 expression per biological replicated (d) is depicted. **e-g,** Quantification of the DMG cells invasion capacity (e) SF8628 treated with FN1 inhibitors RGDS and Avapritinib, (f) DIPG-XII and (g) DIPG XVII treated with FN1 inhibitor RGDS. **h,** Quantification of the microglia cell invasion capability in presence of SF8628 DMG cells and FN1 inhibitors RGDS and Avapritinib. **i,** Quantification of the microglia invasion capability in presence of SF8628 DMG cells after *Fn1* silencing in microglia. **j,** RT-qPCR confirms downregulation of *Fn1* mRNA expression in microglia after *Fn1* silencing. Data are mean ± SEM from 4 (**a, b, e, f, h**) and 3 (**c, d, g, i, j**) independent biological replicates. Statistical annotation * p< 0,05; ** p < 0,001; or exact p-value for indicated comparisons.

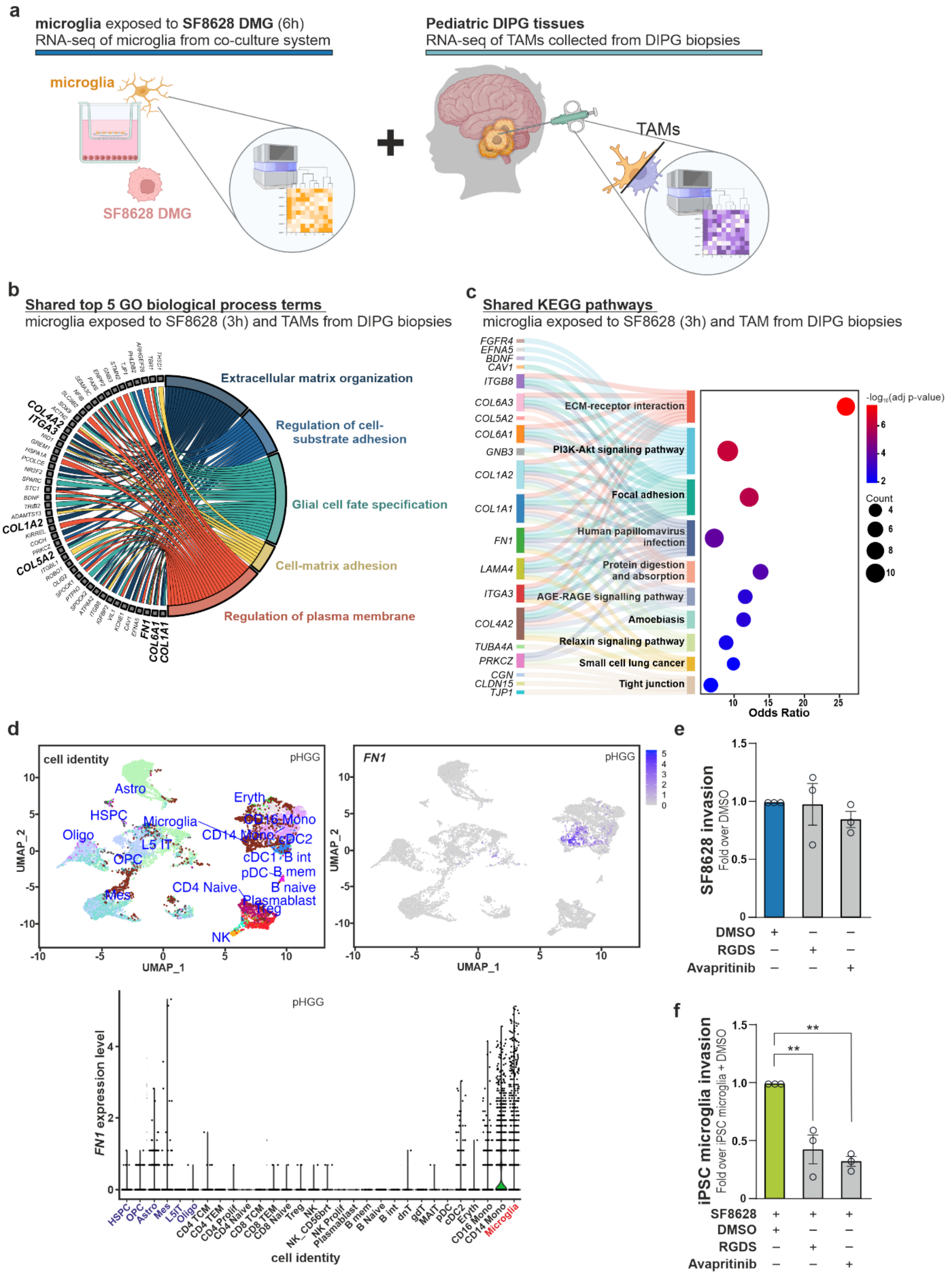

Supplementary Figure 5 | Human DMG H3K27M tumours exhibit an increased expression of tumour-associated myeloid cell-derived ECM components.

a, Illustration representing mutual comparison of transcriptomic data from *in vitro* assayed SF8628 DMG cell stimulated microglia and TAMs from paediatric DMG tumours created with BioRender.com. **b,** Chord diagram representing the top 5 shared GO biological process terms between *in vitro* assayed SF8628 cells-stimulated microglia (3-hour time point) and TAMs from paediatric DMG with highlighted genes involved in extracellular matrix remodulation. **c**, Sankey diagram combined with dot plot representing top 10 significant terms sorted by $-\log_{10}$(p-value) for shared KEGG pathways. Size of circle represents number of genes included in each term, while X-axis represents calculated Odds ratio. **d,** Uniform manifold approximation and projection (UMAP) of 20 pHGG patient tumour samples showing FN1 expression. **e,** Quantification of the SF8628 DMG cell invasion capability treated with FN1 inhibitors RGDS and Avapritinib. **f,** Quantification of the iPSC microglia cell invasion capability in presence of SF8628 DMG cells and FN1 inhibitors RGDS and Avapritinib. Data in **b** and **c** originates from mutual comparison of 3 independent *in vitro* biological replicates of microglia exposed to SF8628 and 5 human DMG biopsies. Data in **c** originates from 20 pHGG patient tumour samples. Human iPSC-related data are represented as mean ± SEM from 3 (**e**, **f**) independent biological replicates. Statistical annotations $^{**}p < 0.01$; for indicated comparisons.

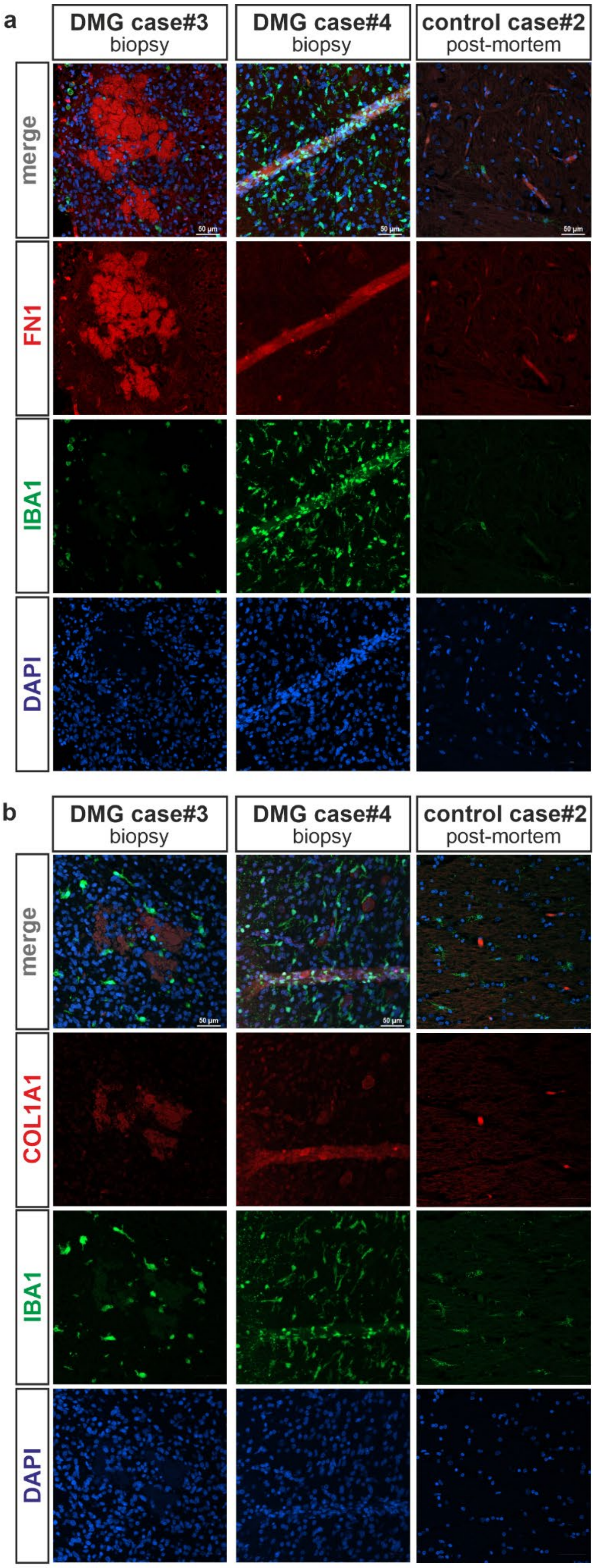
a
DMG case#3
biopsy
DMG case#4
biopsy
control case#2
post-mortem
merge
FN1
IBA1
DAPI
50 µm
b
DMG case#3
biopsy
DMG case#4
biopsy
control case#2
post-mortem
merge
COL1A1
IBA1
DAPI
50 µm

Supplementary Figure 6 | Human DMG H3K27M tumours exhibit an increased FN1 and COL1A1 protein expressions.

a, Confocal microscopy imaging of two human DMG tumours (biopsy samples), and one age-matched brainstem control case (post-mortem sample), with immunofluorescence staining for FN1 and IBA1. **b**, Confocal microscopy imaging of the same cases as in panel a, with immunofluorescence staining for COL1A1 and IBA1. DAPI used as nuclear counterstain, scale bars. 50µm. Representative images originate from 2 independent DMG cases (biopsies) with aged matched healthy subject (post-mortem).

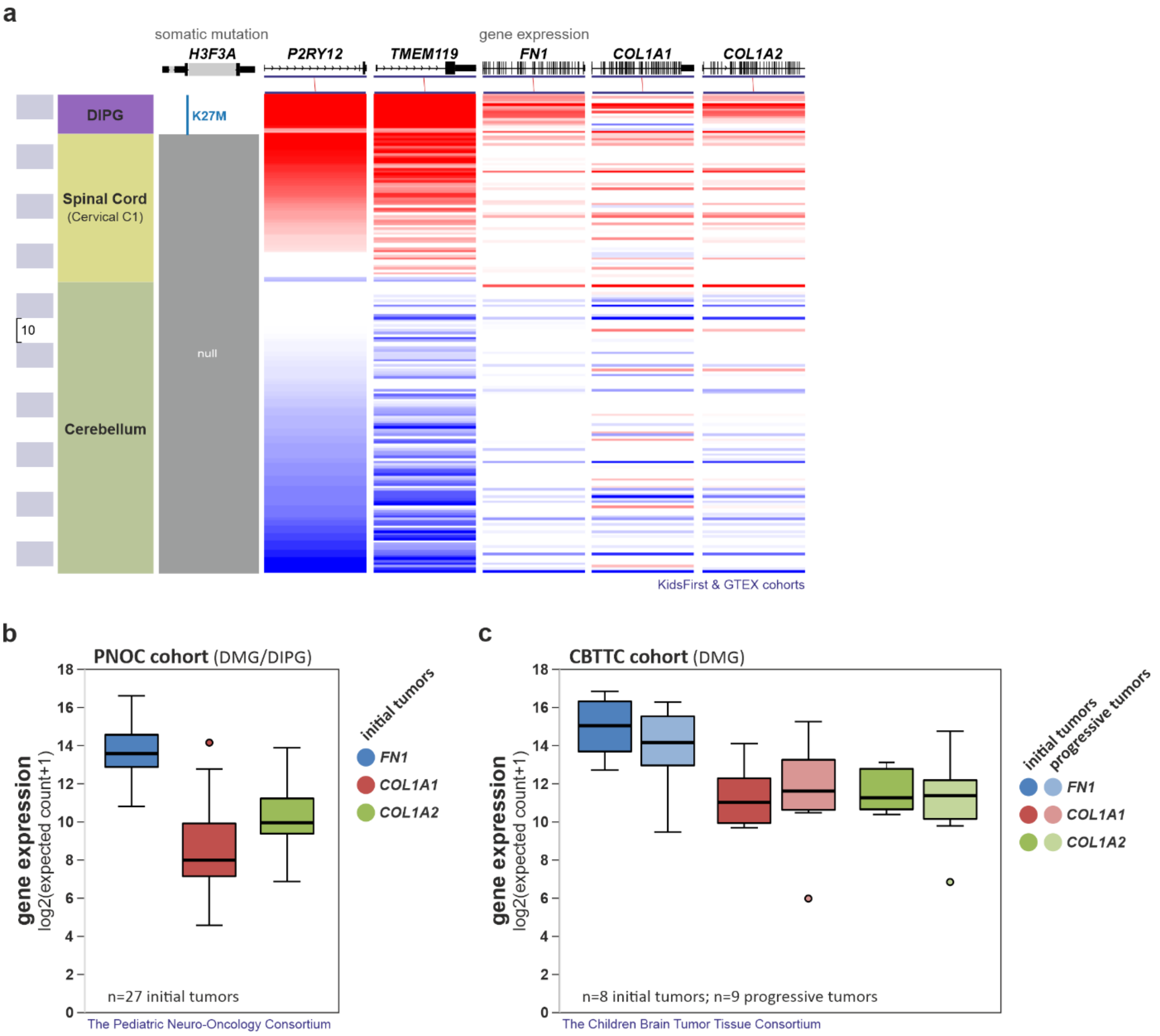

Supplementary Figure 7 | Validation of ECM component expression and prognostic value in DMG H3K27M human cohorts.

a, Illustration of genomic and transcriptomic data extracted from the KidsFirst and the GTEx human cohorts used to assess the expression of *P2RY12*, *TMEM119*, *FN1*, *COL1A1*, and *COL1A2* in tumours from DMG H3K27M subjects as compared to spinal cord, cervical C1 and cerebellum tissues from healthy subjects. **b**, *FN1*, *COL1A1*, *COL1A2* gene expression in initial DMG, H3K27M altered tumours from the Pediatric Neuro-Oncology Consortium (PNOC) human cohort. **c**, *FN1*, *COL1A1*, *COL1A2* gene expression in initial and progressive DMG, H3K27M altered tumours from the Children Brain Tumor Tissue Consortium (CBTTC) human cohort.
