# Supplementary material for: Microglia in diffuse midline glioma contribute to extracellular matrix remodelling and cancer cell invasion
Source: Cell Death Dis. 2026 May 30;17(1):517. doi: 10.1038/s41419-026-08891-y (PMC13222350; doi:10.1038/s41419-026-08891-y)
Supplement: Supplementary file 5 — Original Data [file 41419_2026_8891_MOESM5_ESM.pptx]

## Slide 1
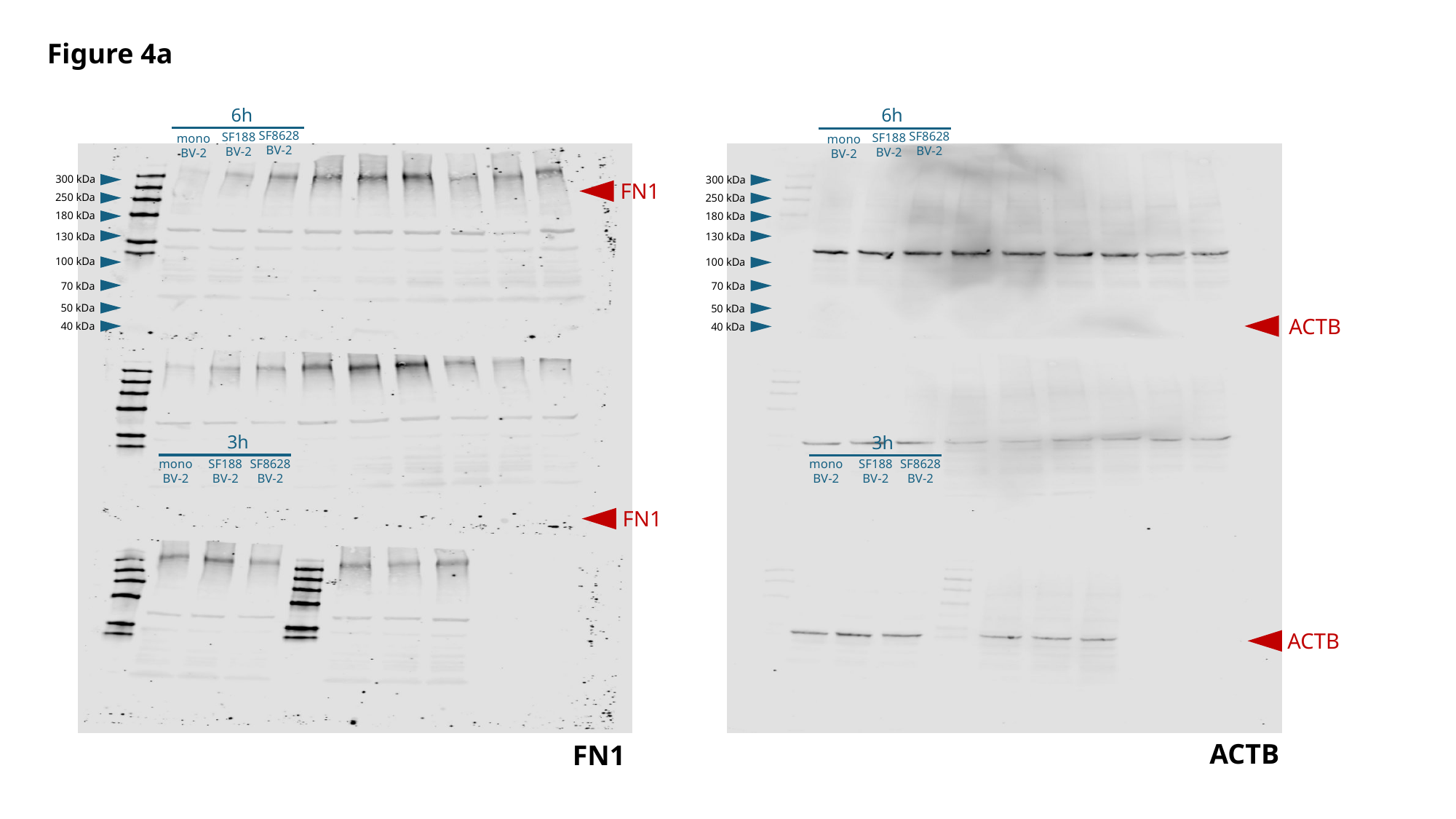

Figure 4a
6h
6h
SF8628
BV-2
SF8628
BV-2
SF188
BV-2
SF188
BV-2
mono
BV-2
mono
BV-2
300 kDa
300 kDa
FN1
250 kDa
250 kDa
180 kDa
180 kDa
130 kDa
130 kDa
100 kDa
100 kDa
70 kDa
70 kDa
50 kDa
50 kDa
ACTB
40 kDa
40 kDa
3h
3h
mono
BV-2
SF188
BV-2
SF8628
BV-2
mono
BV-2
SF188
BV-2
SF8628
BV-2
FN1
ACTB
ACTB
FN1

## Slide 2
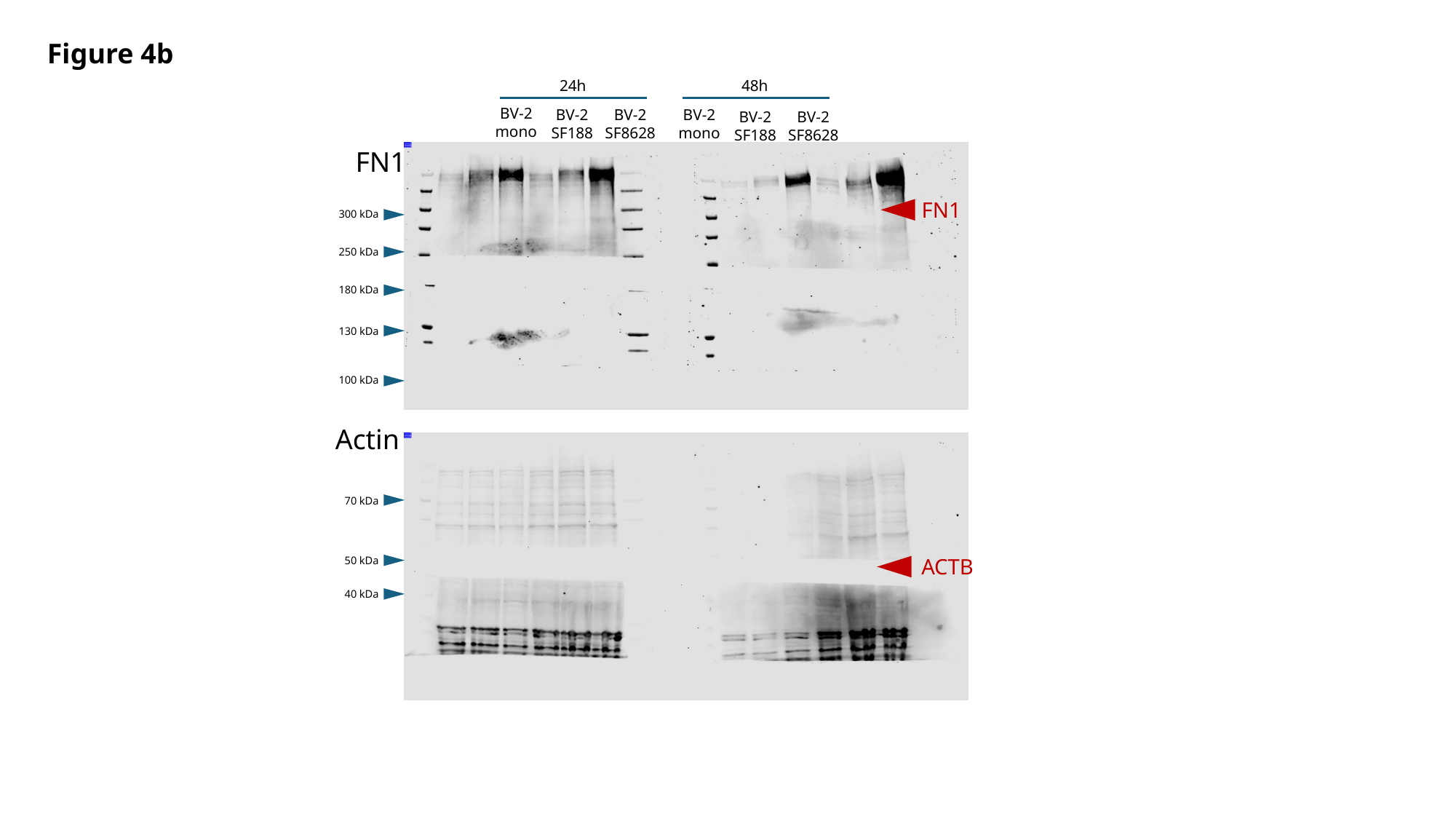

Figure 4b
48h
24h
BV-2
mono
BV-2
SF188
BV-2
SF8628
BV-2
mono
BV-2
SF188
BV-2
SF8628
FN1
FN1
300 kDa
250 kDa
180 kDa
130 kDa
100 kDa
Actin
70 kDa
50 kDa
ACTB
40 kDa

## Slide 3
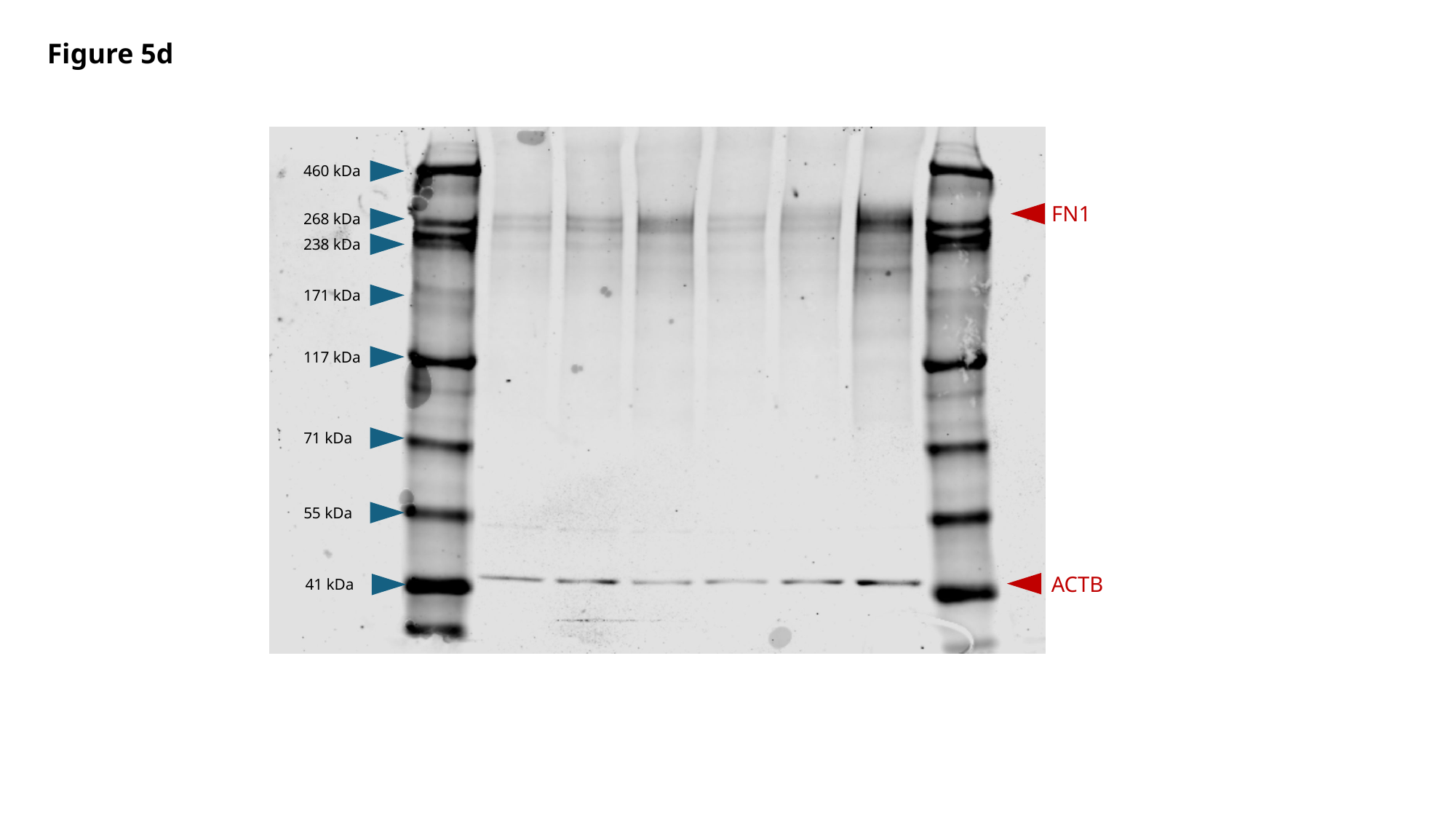

Figure 5d
460 kDa
FN1
268 kDa
238 kDa
171 kDa
117 kDa
71 kDa
55 kDa
ACTB
41 kDa

## Slide 4
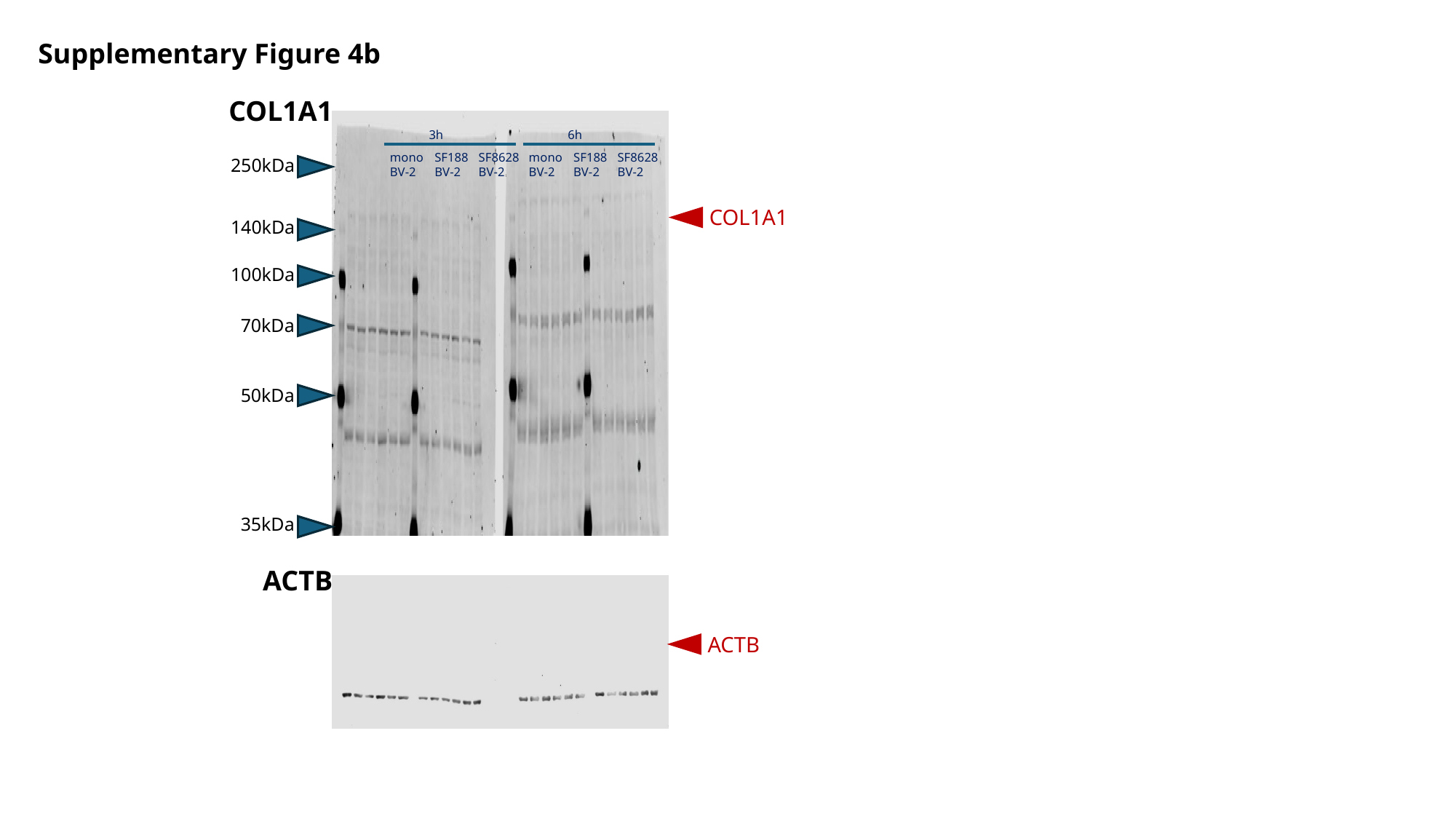

Supplementary Figure 4b
COL1A1
6h
3h
mono
BV-2
SF188
BV-2
SF8628
BV-2
mono
BV-2
SF188
BV-2
SF8628
BV-2
250kDa
COL1A1
140kDa
100kDa
70kDa
50kDa
35kDa
ACTB
ACTB
